# Cox-nnet: An artificial neural network method for prognosis prediction of high-throughput omics data

**DOI:** 10.1371/journal.pcbi.1006076

**Published:** 2018-04-10

**Authors:** Travers Ching, Xun Zhu, Lana X. Garmire

**Affiliations:** 1 Molecular Biosciences and Bioengineering Graduate Program, University of Hawaii at Manoa, Honolulu, HI, United States of America; 2 Epidemiology Program, University of Hawaii Cancer Center, Honolulu, HI, United States of America; University of Cambridge, UNITED KINGDOM

## Abstract

Artificial neural networks (ANN) are computing architectures with many interconnections of simple neural-inspired computing elements, and have been applied to biomedical fields such as imaging analysis and diagnosis. We have developed a new ANN framework called Cox-nnet to predict patient prognosis from high throughput transcriptomics data. In 10 TCGA RNA-Seq data sets, Cox-nnet achieves the same or better predictive accuracy compared to other methods, including Cox-proportional hazards regression (with LASSO, ridge, and mimimax concave penalty), Random Forests Survival and CoxBoost. Cox-nnet also reveals richer biological information, at both the pathway and gene levels. The outputs from the hidden layer node provide an alternative approach for survival-sensitive dimension reduction. In summary, we have developed a new method for accurate and efficient prognosis prediction on high throughput data, with functional biological insights. The source code is freely available at https://github.com/lanagarmire/cox-nnet.

## Author summary

The increasing application of high-througput transcriptomics data to predict patient prognosis demands modern computational methods. With the re-gaining popularity of artificial neural networks, we asked if a refined neural network model could be used to predict patient survival, as an alternative to the conventional methods, such as Cox proportional hazards (Cox-PH) methods with LASSO or ridge penalization. To this end, we have developed a neural network extension of the Cox regression model, called Cox-nnet. It is optimized for survival prediction from high throughput gene expression data, with comparable or better performance than other conventional methods. More importantly, Cox-nnet reveals much richer biological information, at both the pathway and gene levels, by analyzing features represented in the hidden layer nodes in Cox-nnet. Additionally, we propose to use hidden node features as a new approach for dimension reduction during survival data analysis.

## Introduction

With the wide application of genomics technologies, gene expression data of patients are often used as inputs to predict patients’ survival. Computationally, survival prediction is usually framed as a regression problem to model patients’ survival time (or other event time). The most common method is the Cox-PH model, a semi-parametric proportional hazards model, where the covariates of the models explain the relative risks of the patients, termed hazard ratios [1]. Given the large amount of input features in gene expression data, penalization methods such as LASSO (L1 norm), ridge (L2 norm) and MCP [2] regularizations are often used to help select representative feature in Cox-PH models. A modification of Cox-PH model is CoxBoost [3]. It is an iterative “gradient boosting” method, where the parameters are separated into individual partitions. The partition that leads to the largest improvement in the penalized partial log likelihood is selected and in subsequent iterations, the model selects another block and refits those parameters by maximizing the penalized partial log likelihood [3]. Random Forests Survival (RF-S) is a tree-based, non-linear, ensemble method [4], rather than a proportional hazards model. For each tree in the forest, data is bootstrapped, and nodes are split by maximizing the log-rank statistic. The cumulative hazard function (CHF) is estimated in each tree and a patient’s CHF is calculated as an average over all the trees in the ensemble.

Besides these methods above, Artificial Neural networks (ANNs), a type of model that is based on the idea of neurons in processing information, could be trained to predict survival as well. Developed in 1943, ANNs were used to model non-linear behavior [5]. In an ANN, hidden units, termed as neurons or nodes, may be activated or deactivated, depending on the input signals, based their own linear weight and bias parameters. The data are fed forward through the network, and for each hidden unit these weight and bias parameters are learned through back propagation along the gradient of the loss function. In recent years, ANNs have caught renewed attention to solve problems in genomics field [6, 7], thanks to increased parallel computing power and the promise of deep learning [8]. For example, Alipanhi et al. used deep learning in order to better predict the bind of RNA and DNA to proteins [9]. Ciresan et al. used convolutional neural networks to detect cell mitosis in histological breast cancer images [10]. However, relative to these new areas, survival prediction using ANN has been lagging behind.

The first ANN model to predict survival was done by Faraggi and Simon, who used four clinical input parameters to model prostate cancer survival[11]. However, their simple model was not suitable for high throughput input data, where tens of thousands of features are present per patient. Subsequently, other authors attempted to implement ANN methods to predict patient survival. One study applied ANNs to high dimensional survival data by simplifying the regression as a binary classification problem [12, 13], and another study fit continuous variables of survival time to discrete variables through binning [12, 13]. These approaches potentially led to loss of accuracy in prediction. Another study used time as an additional input in order to predict patient survival or censoring status [14], which will overfit when the survival and censoring are correlated. Thus far, an ANN model based on proportional hazards to analyze high throughput data in the genomics era is lacking.

To address all the issues of ANN based predictions as mentioned earlier, we have developed a new software package, named Cox-nnet. We use a two layer neural network: one hidden layer and the output layer. Rather than approximating survival as a classification problem, we used the output layer to perform Cox regression based on the activation levels of the hidden layer. Cox-nnet also computes feature importance scores, so that the relative importance of specific genes to prognosis outcome can be assessed. More importantly, the hidden layer node structure in the ANN can be analyzed to reveal more useful information regarding relevant genes and pathways, compared to other methods in the study. A similar idea for classification (rather than survival analysis) was recently explored in dimension reduction of single cell RNA-Seq data, in which a set of input genes with high weights to the hidden nodes of the neural network, in single cell RNA-Seq was analyzed using GO analysis [15]. Overall, Cox-nnet is a desirable survival analysis method with both excellent predictive power and ability to elucidate biological functions related to prognosis.

## Results

### Cox-nnet structure and optimization

**Fig 1:**
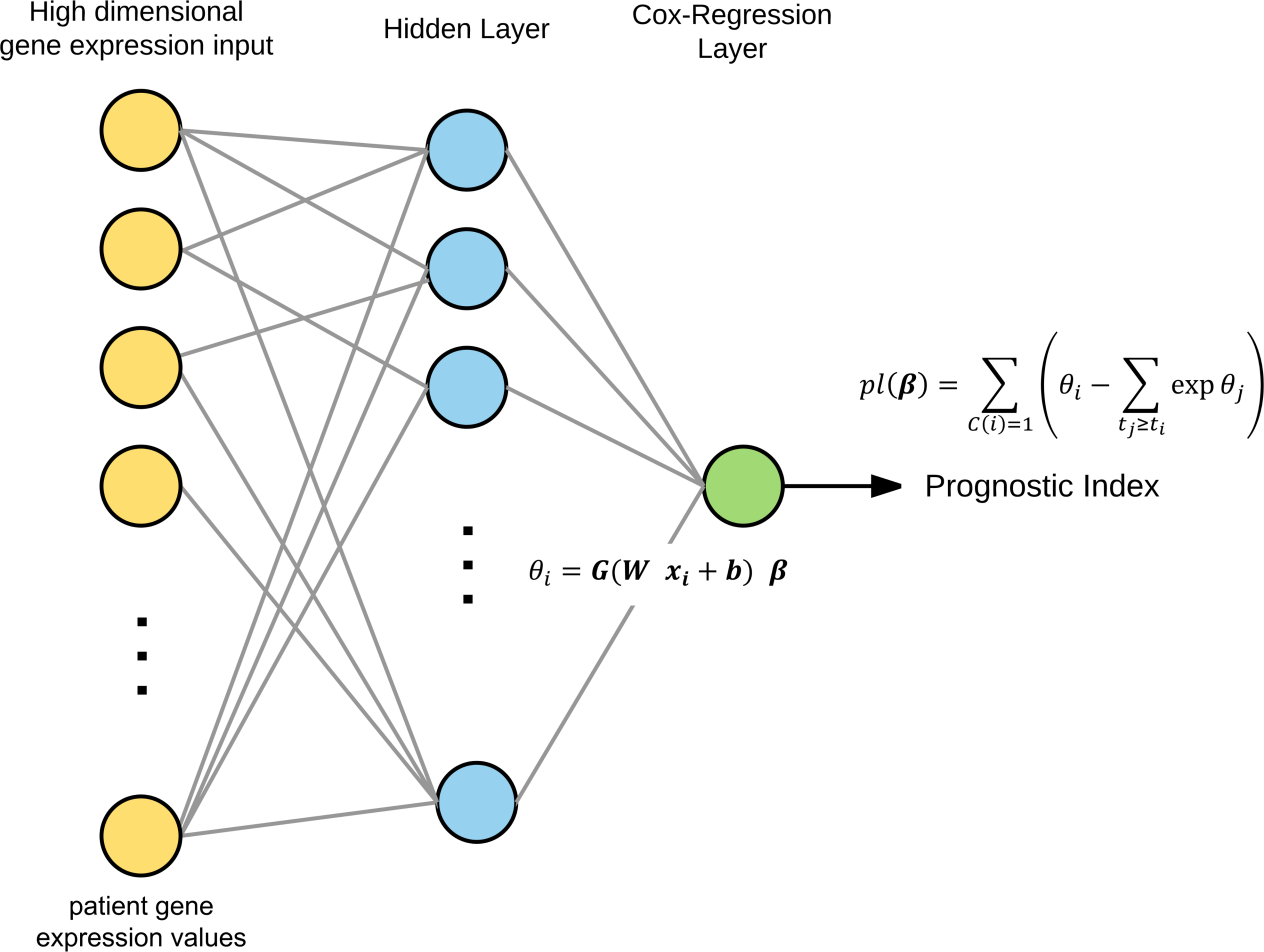
An overview of the optimized Cox-nnet neural network architecture used in this study. Cox-nnet is composed of one hidden layer and an output “Cox-regression” layer. It is optimized to work on high dimensional gene expression data. The model is trained to minimize the partial log likelihood using back-propagation.

The neural network model used in this paper is shown in Fig 1 and an overview of modules in the Cox-nnet package is shown in S1 Fig. The current ANN architecture is composed of the input, one fully connected hidden layer (143 nodes) and an output “proportional hazards” layer. Cox-nnet performs cross-validation (CV) to find the optimal regularization parameter. Due to the large number of parameters, overfitting is a potential problem in ANNs, particularly for small datasets. Thus for regularization, we experimented with a range of regularization methods, including ridge, dropout [16], and the combination of ridge and dropout (see details in Methods). We found that dropout regularization offered overall the best model (S2A and S2B Fig). Furthermore, we compared Cox-nnet structures within no hidden layer (a standard Cox-PH model), one hidden layer (143 nodes) and two hidden layers (143 nodes in both layers) under dropout regularization (S3 Fig). We found that a single hidden layer Cox-nnet performed slightly better than those with no hidden layer (standard Cox-PH) or two hidden layers (S3A and S3B Fig). Thus, we used the single hidden-layer Cox-nnet with dropout regularization (average dropout rate = 7.75 +/- 0.042), for comparison with other survival methods in all following analyses.

Many other functions are implemented to improve the usability of the package (S1 Fig). Among them, the optimizers for adapting the learning rate include momentum gradient descent [17] and Nesterov accelerated gradient [18]. A comparison of these descent methods is shown in S4A Fig. We chose Nesterov accelerated gradient search method for this report. Other parameterization details of Cox-nnet are described in the Methods section.

### Performance comparison of survival prediction methods

We compared four methods, including Cox-nnet, Cox-PH (including Ridge, LASSO and MCP penalizations), CoxBoost and RF-S on 10 datasets from The Cancer Genome Atlas (TCGA). These datasets were selected for having at least 50 death events (S1 Table). For each dataset, we trained the model on 80% of the randomly selected samples and determined the regularization parameter using 5-fold CV on the training set. We evaluated the performance on the remaining 20% holdout test set. We replicated this evaluation 10 times in order to assess the average distribution of each method.

We used four accuracy metrics to evaluate the performance of the model. The first one is C-IPCW (inverse probability of censoring weighted) [19]. This metric aims to overcome the inaccuracy of the unweighted concordance index when censoring time is correlated with the patient’s hazard score. The second metric is Harrell’s concordance index (C-harrel) [20], which is an unweighted concordance index that evaluates the relative ordering of the samples, comparing the prognostic index (i.e., log hazard ratio) of each patient with the survival times. The third metric is the log-ranked p-value from Kaplan-Meier survival curves of two different survival risk groups. This is done by using the median Prognosis Index (PI), the output of Cox-nnet, to dichotomize the patients into high risk and low risk groups, similar to our earlier reports [21, 22]. A log-ranked p-value is then computed to differentiate the Kaplan-Meier survival curves from these two groups. It is worth noticing that the dichotomization of patients ignores the differences within each dichotomized group, thus may lead to less accuracy compared to C-index and IPCW metrics. Finally, the Integrated Brier Score (Brier) was also calculated. This score calculates the squared error between the predicted survival probability and the actual survival of patients at each time point [22–24].

**Fig 2:**
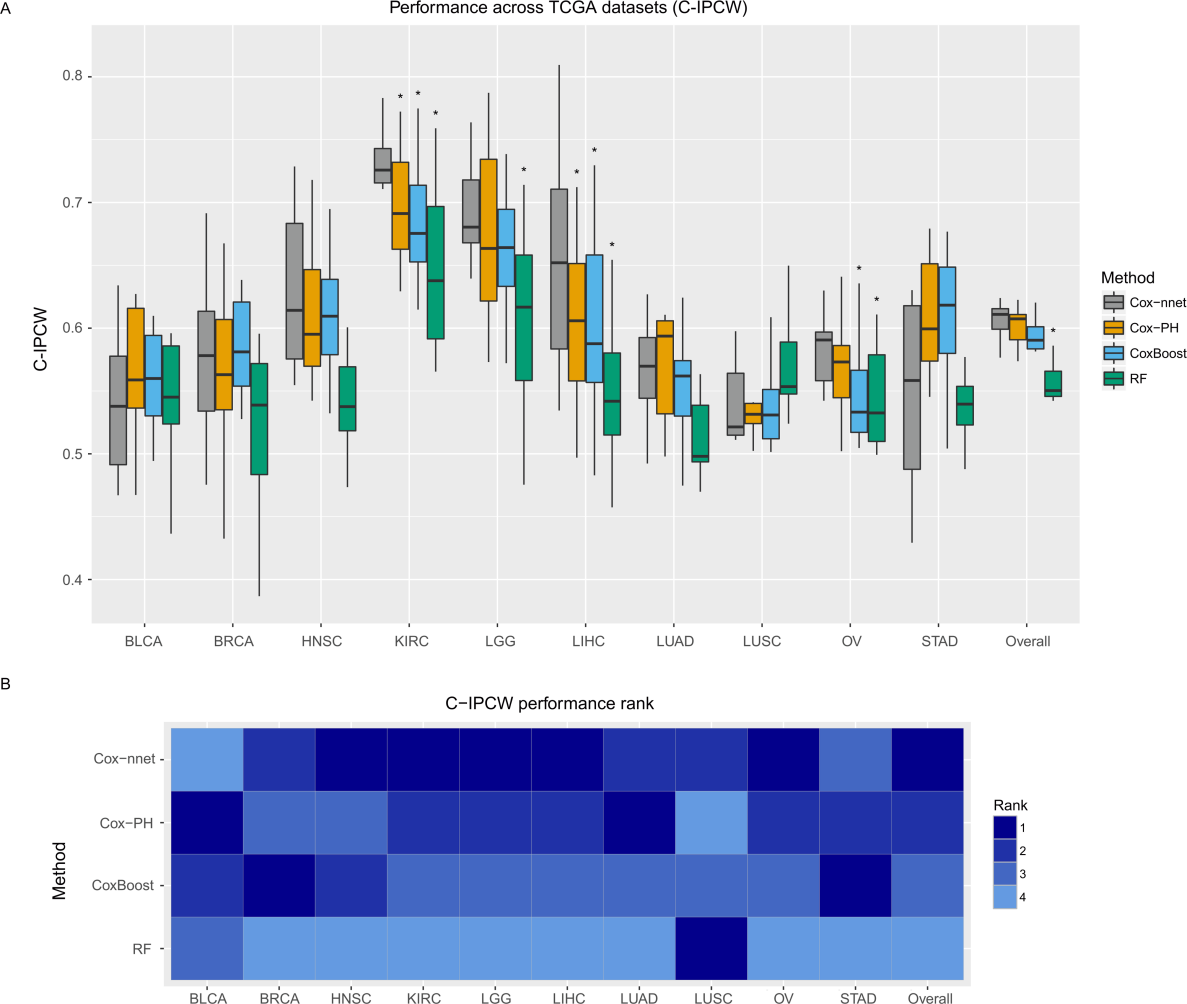
A. Boxplot of the C-IPCW of the 10 TCGA datasets using four prognosis-predicting methods: Cox-nnet (dropout), CoxBoost, Cox-PH (ridge) and RF-S. The data were randomly split into 80% training and 20% testing sets, and repeated 10 times. Average C-IPCWs are presented as the metric. For “overall” condition, all 10 TCGA cancer datasets are combined as one “cancer” dataset. Sign * indicates statistical significance (p < 0.05). B. Heatmap of the performance rank of each dataset, based on the order of the average C-IPCW scores. Ranks 1, 2, 3, and 4 indicate the descending performance of each computational method.

The comparison of C-IPCW among the four methods over the 10 TCGA datasets is shown in Fig 2. Based on the C-IPCW score, Cox-nnet has better overall rankings than other methods (Fig 2B), but the improvement over Cox-PH is lacking statistically significance in most cases (Fig 2A). Note among the three penalization methods applied to Cox-PH, ridge penalization has the best overall accuracy (S5 Fig), and thus Cox-PH with ridge penalization is chosen to compare with the other methods. However, when using C-harrel (S6 Fig) and the log-rank p-value metrics (S7 Fig), Cox-nnet had significantly improved performance compared to all other methods. Based on the Brier score metric, Cox-nnet had significantly higher predictive accuracy compared to RF-S (S8 Fig). Overall, the other non-linear method (RF-S), an ensemble-based method consistently ranks worse than Cox-nnet and Cox-PH (Fig 2, S6, S7 and S8 Figs).

### Hidden layer nodes of Cox-nnet are surrogate prognostic features

**Fig 3:**
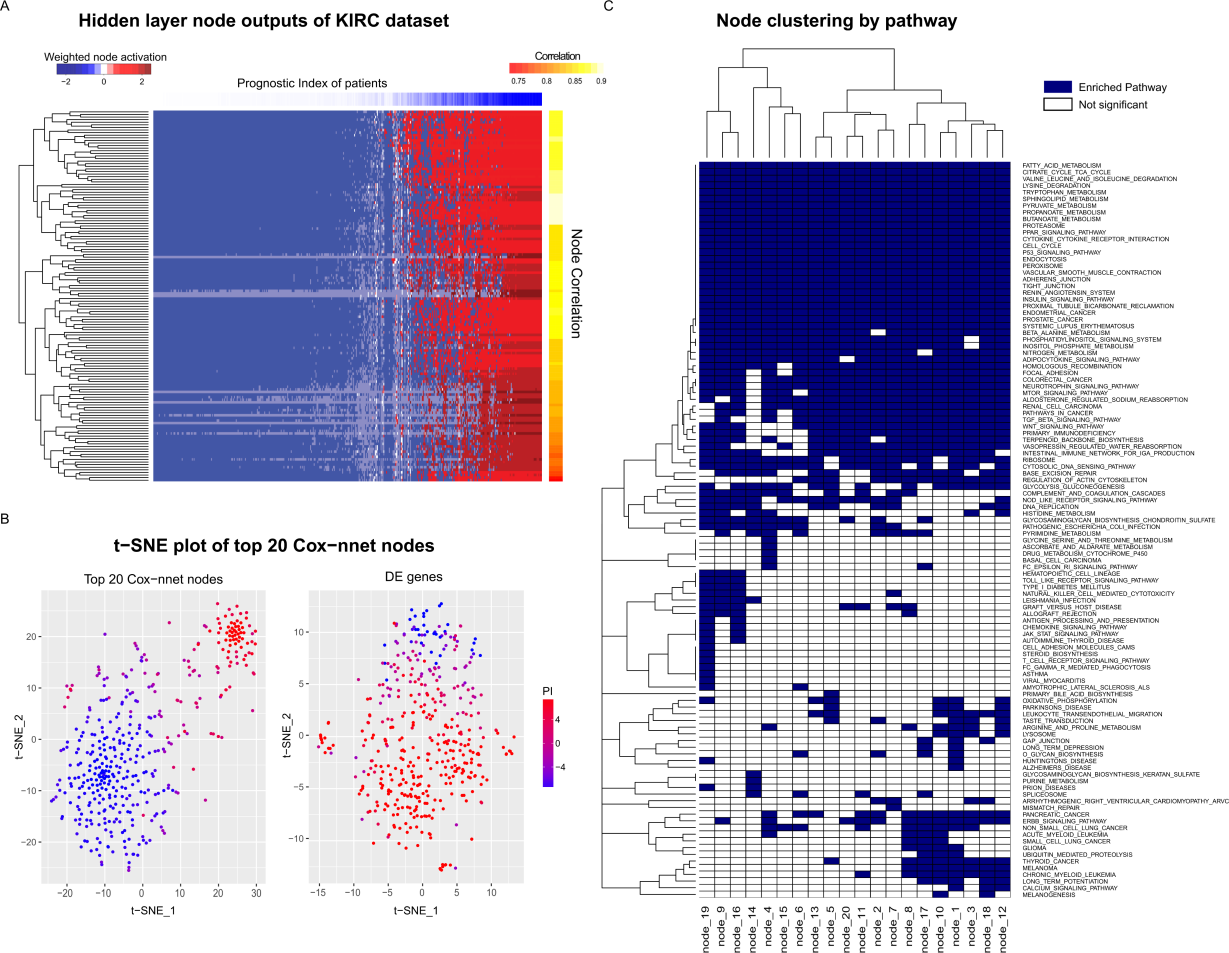
A. Hidden node activation weighted by the corresponding Cox layer coefficients of the TCGA KIRC dataset. The columns represent individual patient scores, ordered by their Prognostic Index. The rows represent the node activations. B. t-SNE plot of the top 20 nodes (left) and t-SNE of differentially expressed genes between the two groups with low and high prognostic index, respecitively (right). C. Gene Set Enrichment Analysis: significantly enriched KEGG pathways of the top 20 hidden nodes (adjusted p-value < 0.05).

To explore the biological relevance of the hidden nodes of Cox-nnet, we used the TCGA Kidney Renal Cell Carcinoma (KIRC) dataset as an example. We first extracted the contribution of each hidden node to the PI score for each patient (Fig 3A). The contribution was calculated as the output value of each hidden node weighted by the corresponding coefficient at the Cox regression output layer. As expected, the value of the hidden nodes strongly correlated to the PI score. However, there is still significant heterogeneity among the nodes, suggesting that individual nodes may reflect different biological processes.

We hypothesize that the top (most variable) nodes may serve as surrogate features to discriminate patient survival. To explore this idea, we selected the top 20 nodes with the highest variances and presented the patients PI scores using t-SNE (Fig 3B). t-SNE is a non-linear dimensionality reduction method that embeds high-dimensional datasets into a low dimensional space (usually two or three dimensions). This method has been widely used to visualize data with large number of features, by enhancing the separation among samples[25]. The hidden nodes represent a dimension reduction of the original data and they clearly discriminate samples by their PI scores, as shown by the t-SNE plot (Fig 3B, left). As comparison, we performed t-SNE using all differentially expressed genes of patients with low prognostic index and high prognostic index (Fig 3B, right). The t-SNE plots demonstrates that the nodes in Cox-nnet effectively capture the survival information. Therefore, the top node PI scores can be used as features for dimension reduction in survival analysis.

### Hidden layer nodes of Cox-nnet are associated with biological functions

To further explore the biological relevance of the top 20 hidden nodes, we conducted Gene Set Enrichment Analysis (GSEA) [26] using KEGG pathways [27], as described in the Methods section. Briefly, we calculated significantly enriched pathways using Pearson’s correlation between the log transformed gene expression input and the output score of each node across all patients in the KIRC dataset (Fig 3C and S2 Table). We compared these enriched pathways to those from GSEA of the Cox-PH (ridge) model (S3 Table), the competing model with the second best prognosis prediction. A total of 110 (out of 187) significantly enriched pathways (S2 Table) were identified in at least one node, including seven pathways enriched in all 20 nodes that were not found by the Cox-PH method (Table 1). In contrast, Cox-PH only identified 30 significantly enriched pathways using the same significance threshold. We also used the genes values from CoxBoost and RF-S, however they did not produce any significantly enriched pathways. Among the seven pathways enriched in all 20 nodes from Cox-nnet, the p53 signaling pathway stands out as an important biologically relevant pathway (S9 Fig), since it was shown to be highly prognostic of patient survival in kidney cancer [28].

**Table 1:** Cox-nnet node-associated pathways. Significantly enriched pathways from common to all 20 hidden nodes that are not found in the Cox-PH Gene Set Enrichment Analysis (Adjusted P < 0.05).

Pathway	P.value	P.adjusted	Nodes
KEGG adherens junction	0.000	0.001	1-20
KEGG endocytosis	0.000	0.001	1-20
KEGG insulin signaling pathway	0.000	0.001	1-20
KEGG lysine degradation	0.000	0.003	1-20
KEGG p53 signaling pathway	0.000	0.003	1-20
KEGG pyruvate metabolism	0.000	0.001	1-20
KEGG sphingolipid metabolism	0.001	0.005	1-20

**Fig 4:**
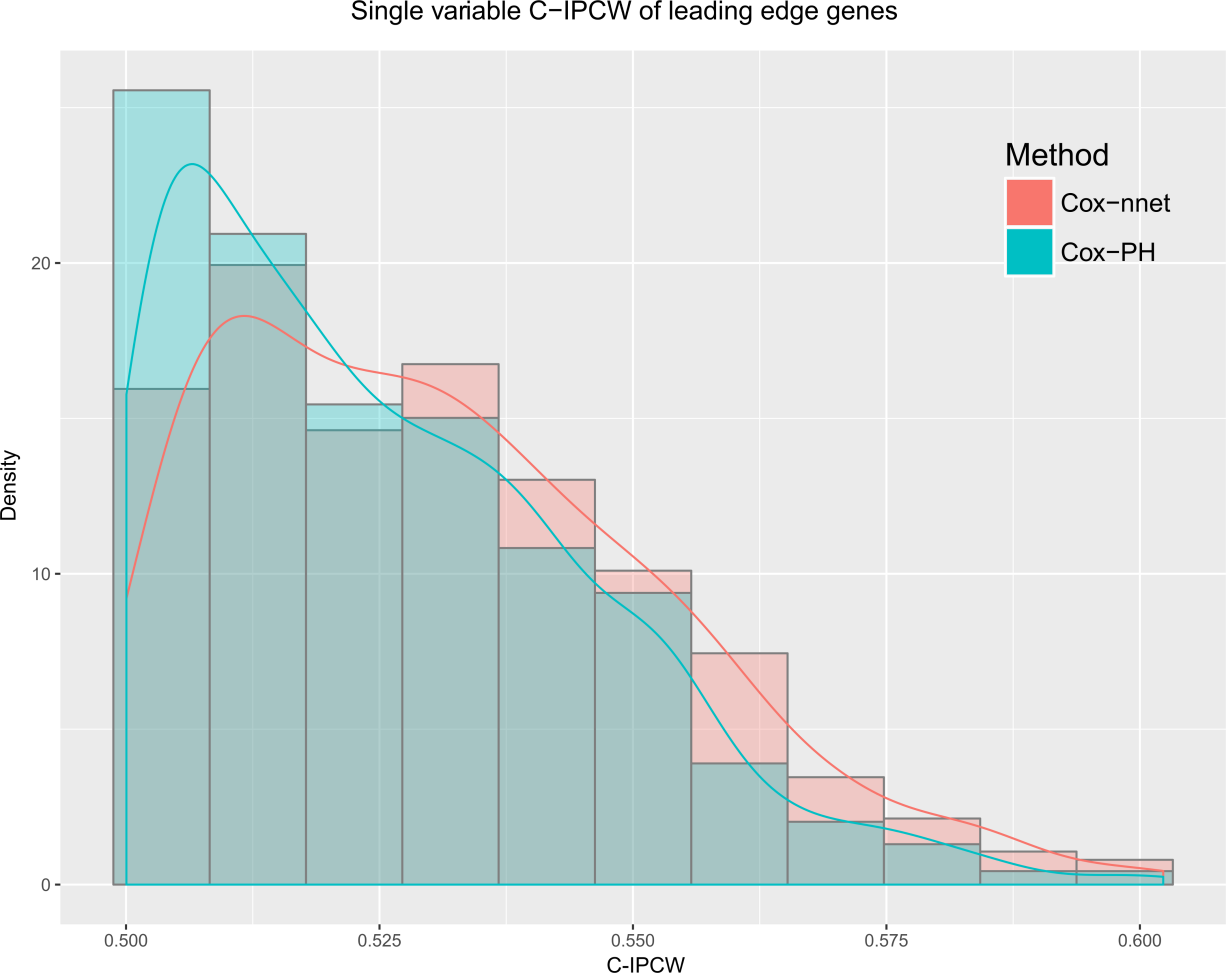
Single variable C-IPCW scores of the leading edge genes from Cox-nnet and Cox-PH. The leading edge genes are obtained using Gene-Set Enrichment Analysis, and they are genes contributing positively to the maximum value of the pathway enrichment score[29]. Cox-nnet has significantly higher C-IPCW scores (p = 1.253e-05).

**Fig 5:**
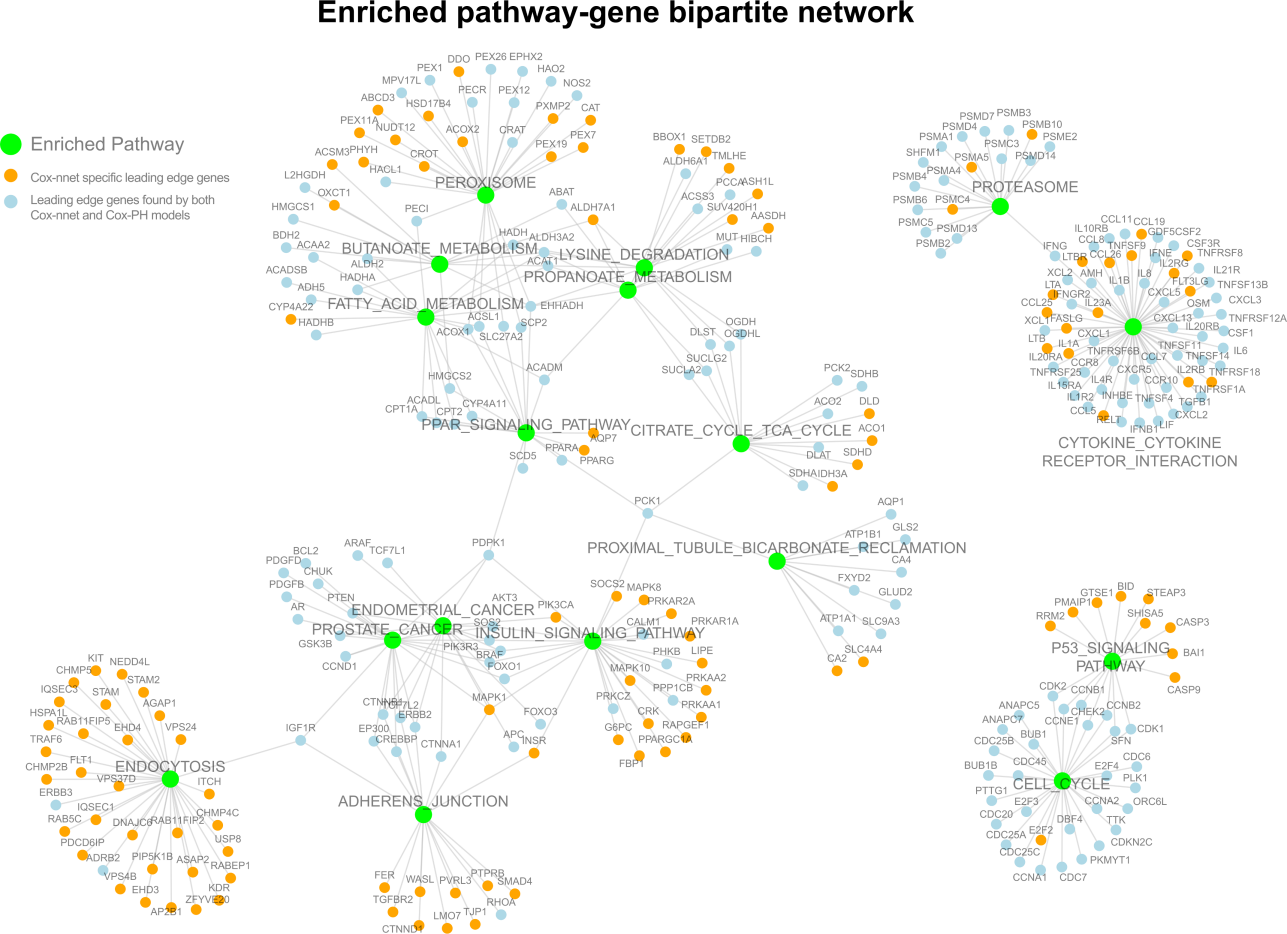
Enriched pathway-gene bipartite network from the leading edge genes and significantly enriched pathways. Significantly enriched pathways common to all 20 hidden nodes are labeled in green. Leading edge genes found uniquely in Cox-nnet are labeled in orange, and genes found in both Cox-nnet and Cox-PH are labeled in blue.

Next, we estimated the predictive accuracies of the leading edge genes [27] enriched in the GSEA from Cox-nnet vs. those enriched in Cox-PH model. Leading edge genes are those genes in the pathway of interest that contribute positively to the enrichment score in GSEA. We used the C-IPCW of each leading edge gene, obtained from single-variable analysis (Fig 4). Collectively, leading edge genes from Cox-nnet have significantly higher C-IPCW scores (p = 1.253e-05) than those from Cox-PH, suggesting that Cox-nnet has selected more informative features. In order to visualize these gene level and pathway level differences between Cox-nnet and Cox-PH, we reconstructed a bipartite graph between leading edge genes for Cox-nnet or feature genes (for Cox-PH) and their corresponding enriched pathways (Fig 5). Besides the p53 pathway mentioned earlier that is specific to Cox-nnet, several other pathways, such as insulin signaling pathway, endocytosis and adherens junction, also have many more genes enriched in Cox-nnet. Among these genes specific to Cox-nnet, many have been previously reported to relevant to renal carcinoma development and prognosis, such as CASP9[27], TGFBR2[30], KDR (VEGFR)[31]. These results suggest that Cox-nnet model reveals richer biological information than Cox-PH.

Additionally, we compared the partial derivative of the hidden nodes (rather than the Cox-nnet output), with respect to the input genes. We first calculated the gradient for each patient and calculated the average partial derivatives and replicated the GSEA analysis as for the previous analysis. However, we found that fewer pathways are significant, and are less relevant to cancer using this approach.

### Evaluation of gene input relative to survival in Cox-nnet

To further examine the importance of each gene relative to the survival outcome, we calculated the average partial derivative of the output of the model (i.e., the log hazard ratio) with respect to each input gene value across all patients. As demonstrated by the leading edge genes in seven common pathways of all nodes in Cox-nnet, the feature importance scores from Cox-nnet appear to be more biologically insightful compared to the feature importance values from the Cox-PH model (S9 Fig). For example, the feature importance for the BAI1 gene in the p53 pathway is much higher in the Cox-nnet model compared to the Cox-PH model. Corresponding to our finding, the BAI gene family was found to be involved in several types of cancers including renal cancer [32]. BAI1 acts as an inhibitor to angiogenesis and is transcriptionally regulated by p53 [33–36]. Its expression level was significantly decreased in tumor vs. normal kidney tissue, and was even lower in advanced stage renal carcinoma[37]. Mice kidney cancer models treated with BAI1 showed slower tumor growth and proliferation [36]. Additionally, the MAPK1 gene (also known as ERK2), annotated in two pathways identified by Cox-nnet (the Adherens Junction and Insulin Signaling pathway), has a much higher feature importance score in Cox-nnet compared to Cox-PH. MAPK1 is one of the key kinases in intra-cellular transduction, and was found constitutively activated in renal cell carcinoma [38]. Drugs inhibiting the MAPK cascade have been targeted for development[39]. We list the top 20 genes from each method in S4 Table.

## Discussion

We have implemented Cox-nnet, a new ANN method, to predict patient survival from high throughput omics data. Cox-nnet is an alternative to the standard Cox-PH regression, enabling automatic discovery of biological features at both the pathway and gene levels. The hidden nodes in the Cox-nnet model have distinct activation patterns, and can serve as surrogate features for survival-sensitive dimension reduction. More significantly enriched KEGG pathways that correlate with top nodes in Cox-nnet are identified, as compared to those from the Cox-PH model, suggesting that Cox-nnet reveals more relevant biological information. We show how a critical pathway for renal cancer development, p53 pathway is identified only by cox-nnet but not Cox-PH model in TCGA KIRC dataset. Other pathways, including insulin signaling pathway, endocytosis and adherens junction, have many more genes enriched by Cox-nnet. Moreover, leading edge genes obtained from these KEGG pathways identified as enriched by Cox-nnet (which are a fraction of the gene features considered by the model) have collectively higher associations with survival. Enrichment analysis on the top genes from Random Forest and CoxBoost did not produce any significant pathways. As a promising new predictive method for prognosis, the current Cox-nnet implementation has some limitations. Its architecture is relatively simple, including one or two hidden layers and an output Cox regression layer. It is possible to incorporate other more sophisticated architecture into the model, such as including more layers of neurons or more sophisticated hidden layers. However, deeper ANN is not necessarily more beneficial (S3 Fig), when compared to the regularization methods. This suggests that ANN may overfit the small size of the genomics data tested. New variations of neural networks, such as convolutional neural network approach or a recurrent network approach as those reported showed good performance in processing imaging or other types of positional data [40], and they could be used as input to a proportional hazards output layer. Additionally, it is possible to embed *a priori* biological pathway information into the network architecture, e.g., by connecting genes in a pathway to a common node in the next hidden layer of neurons [15]. In the future, we plan to further analyze how different neural network architectures affect the performance of Cox-nnet and compare the biological insights from the various models.

## Methods

### Datasets

We analyzed 10 TCGA datasets which were combined into a pan-cancer dataset. The TCGA datasets included the following cancer types: Bladder Urothelial Carcinoma (BLCA), Breast invasive carcinoma (BRCA), Head and Neck squamous cell carcinoma (HNSC), Kidney renal clear cell carcinoma (KIRC), Brain Lower Grade Glioma (LGG), Liver hepatocellular carcinoma (LIHC), Lung adenocarcinoma (LUAD), Lung squamous cell carcinoma (LUSC), Ovarian serous cystadenocarcinoma (OV) and Stomach adenocarcinoma (STAD). RNA-Seq expression and clinical data were downloaded from the Broad Institute GDAC [41]. Overall survival time and censoring information were extracted from the clinical follow-up data. Raw count data were normalized using the DESeq2 R package [17] and then log-transformed. Datasets were selected from TCGA based on the following criteria: > 300 samples with both RNASeq and survival data and > 50 survival events. In total, 5031 patient samples were used (see S1 Table for a patient tabulation by individual dataset).

### Cox-PH, CoxBoost and Random Forest Survival (RF-S) models

Cox-nnet is an extension to the Cox-PH model. Individual hazard, an instantaneous measure of the likelihood of an event, is estimated based on a set of features [17]. The hazard function is:

$$h(t|x_{i})=h_{0}(t)\;{exp\;\theta }_{i}$$ (1)

$$\theta_{i}=x_{i}^{T}\beta$$ (2)

Where ***θ***_***i***_ is the log hazard ratio for patient ***i***. This model uses partial log-likelihood as the cost function

$$pl(\beta )=\sum_{C(i)=1}\left(\theta_{i}-log\sum_{t_{i}\geq t_{j}}exp\left(\theta_{j}\right)\right)$$ (3)

Since gene expression data have tens of thousands in initial features, penalization methods are usually implemented along with Cox-PH. We experimented with 3 penalization methods, namely LASSO (L1 norm), ridge (L2 norm), and mimimax concave penalty (MCP). MCP attempts to moderate the biased large penalty for large coefficients in LASSO [17] (S5 Fig). MCP reduces the regularization for large coefficients and plateaus at a value selected through cross-validation. LASSO and ridge regularization were performed using the Glmnet R package [42] and the MCP regularization was performed with the Ncvreg R package [43].

CoxBoost, is an iterative “gradient boosting” method modified from the Cox-PH model [44]. In CoxBoost, parameters are separated into individual partitions, and the partition that leads to the largest improvement in the penalized partial log likelihood is selected for that iteration. In subsequent boosting iteration, the model selects another block and refits those parameters by maximizing the penalized likelihood function. In this method, the number of boosting iterations is used as the complexity parameter in CoxBoost and optimized via cross-validation (CV).

Random Forests Survival (RF-S) is a tree-based, non-linear, ensemble method [1], rather than a proportional hazards model. For each tree in the forest, samples are boostrapped, and at each node in a tree, features are boostrapped, and the node is split by selecting the feature that maximizes the log-rank statistic. At the leaf nodes, the cumulative hazard function (CHF) is estimated and a patient’s CHF is calculated as an average over all the trees in the ensemble.

### Cox-nnet

Cox-nnet is a neural network whose output layer is a cox regression. In a Cox-nnet model, ***x***_***i***_ in Eq (2) is replaced by the output of the hidden layer, and the linear predictor is:

$$\theta_{i}={G(Wx_{i}+b)}^{T}\beta$$ (4)

Where ***W*** is the coefficient weight matrix between the input and hidden layer with the size H x J, ***b*** is the bias term for each hidden node and ***G*** is the activation function (applied element-wise on a vector).

In this manuscript, the tanh activation function is used:

$$G(z)=\frac{\exp\left(z\right)-exp(-z)}{\exp\left(z\right)+exp(-z)}$$ (5)

Subsequently, the partial log likelihood in Eq (3) is extended by a ridge regularization term:

$$Cost(\beta ,W)=pl(\beta ,W)+\lambda ({\left\|\beta \right\|}_{2}+{\left\|W\right\|}_{2})$$ (6)

In addition to ridge regularization, we also employ dropout regularization (2). This approach has been shown to reduce overfitting and improve performance over other regularization schemes[45]. In dropout, a hyperparameter *p* is the probability of retaining nodes during each iteration of training. I.e., the activation of each node is set to zero with probability *1-p*. The optimal parameter is determined through CV on the training set, using C-index as the performance metric [2]. A complete description of the hyperparameters and optimization approaches for each method is shown in S5 Table. Briefly, we used 5-fold cross-validation and grid search to optimize parameters in Cox-nnet.

In neural networks, because of the large amount of parameters and hyperparameters, overfitting is a potential problem. In Cox-nnet, we experimented with three regularization approaches given previous guidelines: ridge, dropout and combination of ridge and dropout. Ridge regularization is one of the most common methods to reduce overfitting, recommended by Demuth et al. [3]. In this scheme, the L2 norm of all the weights are added to the cost function of the model, leading to a “weight decay” term in the gradient. Dropout is a recent regularization method for networks, inspired by Bayesian analysis on weighted averages of different network architectures to improve the model performance [4]. In dropout networks, each training iteration uses different network architecture; nodes are randomly deactivated from the network during training based on a probability hyperparameter between 0 and 1. Instead of entire models being reweighted, the output of each node is reweighted during evaluation. This method was previously shown to perform better than other regularization methods, such as ridge regularization [17]. Our results on Cox-nnet confirmed this earlier conclusion. Also similar to a previous study, we found that additional complexity of combining dropout and ridge regularization does not yield better performance [17].

### Implementation of Cox-nnet

We implement Cox-nnet in Python with Theano package, a package for automatic differentiation that is widely used for designing neural networks. Cox-nnet is trained through back propagation. The partial log likelihood is usually written as two summations (one nested within the other) conditioned on survival time and censoring status (Eq 3). Because the partial log likelihood is usually written as a nested summation, one may write a program to calculate the partial log likelihood using nested loops. However, these types of operations are very slow in Theano, whereas matrix operations are very fast. Therefore, it is imperative to convert the usual form of the partial log likelihood into a mathematically equivalent form using matrix multiplication. First, we define an indicator matrix ***R*** with elements:

$$R_{ij}=\left\{ \begin{array}{c} 1,\;t_{i}\leq t_{j}\\ 0,\;t_{i}>t_{j} \end{array}\right.$$ (7)

We also define an indicator vector ***C*** with elements given by the censoring of each patient. An operation using ***R*** replaces the conditional sum over *t*_*i*_ ≥ *t*_*j*_, and an operation using ***C*** replaces the conditional sum over ***C***(***i***) = **1** in Eq 4. For the models trained in this manuscript, the number of iterations was fixed at 1e4. The learning rate was initialized at 0.1, and decayed exponentially by a factor of 0.9 if the loss did not decrease. The number of hidden nodes in the hidden layer is chosen to be the square root of the number of input nodes, following the “pyramid” rule of thumb [21]. The optimization strategy used was Nesterov accelerated gradient [41]. For the two hidden layer neural network, we used the same number of hidden nodes as the single hidden layer model in both the first and second hidden layers.

Many functions are implemented to make it easier to train and evaluate models for survival analysis, including CVSearch, CVProfile, CrossValidation, and TrainCoxMlp (S1 Fig). CVSearch, CVProfile, CrossValidation are methods that perform CV to find the optimal regularization parameter. TrainCoxMlp performs optimization of coefficients on the regularized partial log likelihood function.

The source code of cox-nnet can be found at: https://github.com/lanagarmire/cox-nnet, and can be installed through the Python Package Index (PyPI). Documentation of package can be found at http://garmiregroup.org/cox-nnet/docs/. An example of analyzing Cox-nnet through R can be found at: http://garmiregroup.org/cox-nnet/docs/examples/#interfacing-and-analysis-with-r

Cox-nnet can be run on multiple threads or a Graphics Processing Unit (GPU), an advantage of the Theano framework. We measured the running time on the KIRC dataset and re-measured it 5 times. The computational time for Cox-nnet included model compilation time and cross validation time to optimize dropout.

### Model evaluation

To evaluate the performance of all methods, we resampled the data 10 times. In each resampling iteration, we trained each model on 80% of the samples for each dataset (chosen randomly) and evaluated the performance on the 20% holdout test set. The output of Cox-PH, Cox-nnet and CoxBoost are the log hazard ratios (i.e., Prognosis Index, or PI) for each patient. The hazard ratio describes the relative risk of a patient compared to a non-parametric baseline. In contrast, the output of RF-S is an estimation of the survival time for each patient. We use C-index, IPCW [17], log-ranked p-value and Brier score to measure the accuracy performance of each model. We also compare running time of each model (S4B Fig).

C-index: is a measure of how well the model prediction corresponds to the ranking of the survival data [17]. It is calculated for censored survival data, which evaluates a value between 0 and 1, with 0.5 equivalent to a random process. The C-index can be computed as a summation over all events in the dataset, where patients with a higher survival time and lower log hazard ratios (and conversely patients with a lower survival time but higher log hazard ratios) are considered concordant.

C-IPCW (Inverse Probability of Censoring Weighting): it is a method that takes into account the censoring probability in the concordance index, by weighting pairs of patients in the calculation based on the inverse of their individual probabilities to be censored [17]. In this manuscript, we use the Kaplan-Meier estimate of censorship.

log-ranked p-value: a PI cutoff threshold is used to dichotomize the patients in the data set into higher and lower risk groups, similar to our earlier report [46]. A log-ranked p-value is then computed to differentiate the Kaplan-Meier survival curves between the higher vs. lower risk groups. In this report, we used the median log hazard ratio as the cutoff threshold.

Brier-score: the Brier-score is the mean square error of the difference between the probability of an event and the event value (1 or 0). The integrated Brier score was used as the performance metric, which is the Brier score averaged over the time interval of the dataset.

Running-time: Cox-nnet can be run on multiple threads or a Graphics Processing Unit (GPU), an advantage of the Theano framework. We measured the running time on the KIRC dataset, and re-measured it 5 times. The computational time for Cox-nnet included model compilation time and cross validation time to optimize dropout.

### Feature evaluation

For computing the importance of a feature in Cox-nnet, we use a method of partial derivatives [19]. For each patient, we compute the partial derivatives of each linear output of the model (e.g., the log hazard ratio) with respect to the input. The average of the partial derivatives over each input across all patient samples is calculated as the feature score. As comparison, we also compute the partial derivatives of each hidden layer node with respect to the inputs.

### t-SNE clustering

T-distributed stochastic neighbor embedding (t-SNE) is a non-linear dimensionality reduction method that is commonly used for visualizing high-dimensional data [20]. We selected the top 20 nodes of the Cox-nnet model with the highest variances, and clustered the patient samples using t-SNE. To do this, we used the tsne package in R [47]. As comparison, we also plot t-SNE based on the top 33% and bottom 33% of patients as determined by the prognostic index. A total of 8467 and 5805 genes were deemed significantly up and down regulated respectively in the KIRC dataset, using DESeq2 for differential expression analysis.

### Gene Set Enrichment Analysis (GSEA)

We performed GSEA on the correlation of normalized log gene expression to the node output (for Cox-nnet) or the model output (for Cox-PH, Random Forest and CoxBoost), across all patients in the KIRC dataset. Using the fgsea package in R [48], we calculated statistical significance of the KEGG pathways by performing 10,000 permutations, followed by multiple hypothesis testing with Benjamini Hochberg adjustment.

### Statistical testing between model performances

To test for statistical significance between the performance of Cox-nnet and other methods, we use the “multcomp” package in R to perform multiple linear hypothesis testing, with the method as the factor of interest, and the iteration number (used to control the random sampling seed in each 80%/20% split) as a covariate. We then perform multiple comparisons that compare Cox-nnet with the other methods, and adjust for multiple hypothesis testing with the Benjamini Hochberg procedure [20]. We apply this statistical approach to all performance metrics (C-harrell, C-IPCW, log-rank, and brier score).

### Simulation

overfitting in Cox-nnet may come from patient censoring. To investigate this, we ran a simulation RNA-Seq dataset. We used the ssizeRNA package in R to generate simulated RNA-Seq data counts in R [23, 24]. We generated four sub-groups of 200 patients each (a total of 800 patient samples) with 1000 genes, among which 20% of the genes are differentially expressed for each group. The prognosis index for patients in each group was randomly generated based on expression of 100 randomly selected genes, and the survival times were sampled based on the Weibull survival distribution. Censoring times were chosen from the exponential distribution with rate = 0.05. We randomly generated this dataset 100 times and estimated the performance metrics on 20% holdout test-sets. We compared C-index and IPCW metrics with censoring to uncensored C-index (S10 Fig). Neural network-based Cox-nnet and tree-based Random Forest survival do not differ significantly from Cox-PH models. This may be due to the simplification in the simulated data. For example, the simulated gene-expression does not have common biological functions and embedded co-linearability as shown in TCGA data.

## Supporting information

S1 Fig Overview of the structure, methods and classes in the Cox-nnet package. The arrows in the workflow point from each module to the output of that module. “Model Predictions” and “Var. Importance Score” are the output of Cox-nnet package.
(PDF)

S2 Fig A. Boxplot of C-IPCW of the 10 TCGA datasets among various penalization approaches in Cox-nnet one hidden layer (ridge, drop-out and ridge combined with dropout). Cox-nnet with Ridge and Dropout is optimized based on 5-fold cross-validation. Cox-nnet parameterizations with Ridge and Dropout are optimized based on a single validation set. Each dataset is randomly split into 80% training and 20% testing sets and resampled 10 times to calculate the average performance of each approach. B. Performance rank of each regularization approach, ordered by their average performance in each dataset.
(PDF)

S3 Fig A. Boxplot of C-IPCW of the 10 TCGA datasets comparing zero, one and two hidden layers. Each model was optimized with 5-fold cross-validation. Each dataset is randomly split into 80% training and 20% testing sets and resampled 10 times to calculate the average performance of each approach. B. Performance rank of each regularization approach, ordered by the average C-IPCW scores in each dataset. *: P < 0.05.
(PDF)

S4 Fig A: comparison of descent methods on the TCGA KIRC dataset. The change in cost function is evaluated over 100,000 iterations for three methods: gradient descent, momentum gradient descent and the Nesterov accelerated gradient. B: Boxplots comparing Cox-nnet (CPU and GPU), Cox-PH, Cox-boost and Random Forest (RS-F) running time on the same dataset.
(PDF)

S5 Fig A. Boxplot of C-IPCW of the 10 TCGA datasets comparing Cox-PH regularization methods (LASSO, Ridge and MCP). Each model is optimized with 5-fold cross-validation. Each dataset is randomly split into 80% training and 20% testing sets and resampled 10 times to calculate the average performance of each approach. B. Performance rank of each regularization approach, ordered by the average C-IPCW scores in each dataset. *: P < 0.05.
(PDF)

S6 Fig Boxplot of the C-harrel of the 10 TCGA datasets using four prognosis-predicting methods: Cox-nnet (dropout), CoxBoost, Cox-PH (ridge) and RF-S. The data are randomly split into 80% training and 20% testing sets, and repeated 10 times to calculate the average C-harrel values in each approach. *: P < 0.05.
(PDF)

S7 Fig A. Bar plots of Log-rank p-values of the 10 TCGA datasets. The log rank p-values are calculated first splitting the patients by median prognostic index in the testing data set, in order to compare the survival distributions between the high and low risk groups. The data are randomly split into 80% training and 20% testing sets, and repeated 10 times to calculate the average log-rank p-values in each approach. *: P < 0.05. B. Kaplan-Meier plots from one exemplary repeat showing survival differences between the high and low risk groups. Note that due to dichotomization, log-rank p-values vary much widely compared to other performance metrics.
(PDF)

S8 Fig Boxplot of the Brier score of the 10 TCGA datasets using four prognosis-predicting methods: Cox-nnet (dropout), CoxBoost, Cox-PH (ridge) and RF-S. The data are randomly split into 80% training and 20% testing sets and repeated 10 times to calculate the average Brier scores in each approach. *: P < 0.05.
(PDF)

S9 Fig Variable importance of the common leading edge genes of enriched KEGG pathways. (PDF)

S10 Fig RNA-Seq survival simulation results showing the performance over 100 simulated datasets comparing the C-index, IPCW metric and the uncensored concordance. (PDF)

S1 Table Tabulation of TCGA patients by individual dataset. Event = 1 indicates death.
(XLSX)

S2 Table Significantly enriched pathways from the Cox-nnet method (P < 0.05). (PDF)

S3 Table Significantly enriched pathways from the Cox-PH method (P < 0.05). (PDF)

S4 Table Top 20 genes from the four survival methods compared. (XLSX)

S5 Table Description of hyperparameters in each method and their method of optimization. (XLSX)

## References

[1] Therneau TM, Grambsch PM. Modeling survival data: extending the Cox model: Springer Science & Business Media; 2000.

[2] Breheny P, Huang J. Coordinate descent algorithms for nonconvex penalized regression, with applications to biological feature selection. The annals of applied statistics. 2011;5(1):232 doi: 10.1214/10-AOAS388 22081779 10.1214/10-AOAS388 PMC3212875

[3] Binder H. CoxBoost: Cox models by likelihood based boosting for a single survival endpoint or competing risks. R package version. 2013;1.

[4] Ishwaran H, Kogalur UB, Blackstone EH, Lauer MS. Random survival forests. The Annals of Applied Statistics. 2008:841–60.

[5] McCulloch WS, Pitts W. A logical calculus of the ideas immanent in nervous activity. The bulletin of mathematical biophysics. 1943;5(4):115–33. 2185863

[6] LeCun Y, Bengio Y, Hinton G. Deep learning. Nature. 2015;521(7553):436–44. doi: 10.1038/nature14539 26017442 10.1038/nature14539

[7] Ching T, Himmelstein DS, Beaulieu-Jones BK, Kalinin AA, Do BT, Way GP, et al Opportunities And Obstacles For Deep Learning In Biology And Medicine. bioRxiv. 2017:142760. 10.1098/rsif.2017.0387 PMC5938574 29618526

[8] Jones N. The learning machines Nature Publishing Group MACMILLAN BUILDING, 4 CRINAN ST, LONDON N1 9XW, ENGLAND; 2014.

[9] Alipanahi B, Delong A, Weirauch MT, Frey BJ. Predicting the sequence specificities of DNA-and RNA-binding proteins by deep learning. Nature biotechnology. 2015;33(8):831–8. doi: 10.1038/nbt.3300 26213851 10.1038/nbt.3300

[10] Cireşan DC, Giusti A, Gambardella LM, Schmidhuber J, editors. Mitosis detection in breast cancer histology images with deep neural networks. International Conference on Medical Image Computing and Computer-assisted Intervention; 2013: Springer. 10.1007/978-3-642-40763-5_51 24579167

[11] Faraggi D, Simon R. A neural network model for survival data. Statistics in medicine. 1995;14(1):73–82. 7701159 10.1002/sim.4780140108

[12] Petalidis LP, Oulas A, Backlund M, Wayland MT, Liu L, Plant K, et al Improved grading and survival prediction of human astrocytic brain tumors by artificial neural network analysis of gene expression microarray data. Molecular cancer therapeutics. 2008;7(5):1013–24. doi: 10.1158/1535-7163.MCT-07-0177 18445660 10.1158/1535-7163.MCT-07-0177 PMC2819720

[13] Chi C-L, Street WN, Wolberg WH, editors. Application of artificial neural network-based survival analysis on two breast cancer datasets. AMIA Annual Symposium Proceedings; 2007: American Medical Informatics Association. PMC2813661 18693812

[14] Joshi R, Reeves C, editors. Beyond the Cox model: artificial neural networks for survival analysis part II. Proceedings of the eighteenth international conference on systems engineering; 2006.

[15] Lin C, Jain S, Kim H, Bar-Joseph Z. Using neural networks for reducing the dimensions of single-cell RNA-Seq data. Nucleic Acids Res. 2017;45(17):e156 doi: 10.1093/nar/gkx681 28973464 10.1093/nar/gkx681 PMC5737331

[16] Al-Rfou R, Alain G, Almahairi A, Angermueller C, Bahdanau D, Ballas N, et al Theano: A Python framework for fast computation of mathematical expressions. arXiv preprint arXiv:160502688. 2016.

[17] Srivastava N, Hinton GE, Krizhevsky A, Sutskever I, Salakhutdinov R. Dropout: a simple way to prevent neural networks from overfitting. Journal of Machine Learning Research. 2014;15(1):1929–58.

[18] Qian N. On the momentum term in gradient descent learning algorithms. Neural networks. 1999;12(1):145–51. 12662723 10.1016/s0893-6080(98)00116-6

[19] Bengio Y, Boulanger-Lewandowski N, Pascanu R, editors. Advances in optimizing recurrent networks. Acoustics, Speech and Signal Processing (ICASSP), 2013 IEEE International Conference on; 2013: IEEE.

[20] Gerds TA, Kattan MW, Schumacher M, Yu C. Estimating a time‐dependent concordance index for survival prediction models with covariate dependent censoring. Statistics in Medicine. 2013;32(13):2173–84. doi: 10.1002/sim.5681 23172755 10.1002/sim.5681

[21] Koziol JA, Jia Z. The concordance index C and the Mann–Whitney parameter Pr (X> Y) with randomly censored data. Biometrical Journal. 2009;51(3):467–74. doi: 10.1002/bimj.200800228 19588452 10.1002/bimj.200800228

[22] Wei R, De Vivo I, Huang S, Zhu X, Risch H, Moore JH, et al Meta-dimensional data integration identifies critical pathways for susceptibility, tumorigenesis and progression of endometrial cancer. Oncotarget. 2016. 10.18632/oncotarget.10509 PMC5342415 27409342

[23] Huang S, Yee C, Ching T, Yu H, Garmire LX. A Novel Model to Combine Clinical and Pathway-Based Transcriptomic Information for the Prognosis Prediction of Breast Cancer. PLoS computational biology. 2014;10(9):e1003851 doi: 10.1371/journal.pcbi.1003851 25233347 10.1371/journal.pcbi.1003851 PMC4168973

[24] Huang S, Chong N, Lewis NE, Jia W, Xie G, Garmire LX. Novel personalized pathway-based metabolomics models reveal key metabolic pathways for breast cancer diagnosis. Genome medicine. 2016;8(1):1. 27036109 10.1186/s13073-016-0289-9 PMC4818393

[25] Graf E, Schmoor C, Sauerbrei W, Schumacher M. Assessment and comparison of prognostic classification schemes for survival data. Statistics in medicine. 1999;18(17‐18):2529–45. 10474158 10.1002/(sici)1097-0258(19990915/30)18:17/18<2529::aid-sim274>3.0.co;2-5

[26] Lvd Maaten, Hinton G. Visualizing data using t-SNE. Journal of Machine Learning Research. 2008;9(Nov):2579–605.

[27] Subramanian A, Tamayo P, Mootha VK, Mukherjee S, Ebert BL, Gillette MA, et al Gene set enrichment analysis: a knowledge-based approach for interpreting genome-wide expression profiles. Proceedings of the National Academy of Sciences. 2005;102(43):15545–50. 10.1073/pnas.0506580102 PMC1239896 16199517

[28] Sergushichev A. An algorithm for fast preranked gene set enrichment analysis using cumulative statistic calculation. bioRxiv. 2016:060012.

[29] Girgin C, Tarhan H, seyin u, Hekimgil M, Sezer A, Gürel G. P53 mutations and other prognostic factors of renal cell carcinoma. Urologia internationalis. 2001;66(2):78–83. doi: 10.1159/000056575 11223748 10.1159/000056575

[30] Marques I, Teixeira AL, Ferreira M, Assis J, Lobo F, Maurício J, et al Influence of survivin (BIRC5) and caspase-9 (CASP9) functional polymorphisms in renal cell carcinoma development: a study in a southern European population. Molecular biology reports. 2013;40(8):4819–26. doi: 10.1007/s11033-013-2578-3 23645041 10.1007/s11033-013-2578-3

[31] Akhurst RJ, Derynck R. TGF-β signaling in cancer–a double-edged sword. Trends in cell biology. 2001;11(11):S44–S51. 11684442 10.1016/s0962-8924(01)02130-4

[32] Choueiri TK, Vaishampayan U, Rosenberg JE, Logan TF, Harzstark AL, Bukowski RM, et al Phase II and biomarker study of the dual MET/VEGFR2 inhibitor foretinib in patients with papillary renal cell carcinoma. Journal of Clinical Oncology. 2013;31(2):181–6. doi: 10.1200/JCO.2012.43.3383 23213094 10.1200/JCO.2012.43.3383 PMC3532390

[33] Cork SM, Van Meir EG. Emerging roles for the BAI1 protein family in the regulation of phagocytosis, synaptogenesis, neurovasculature, and tumor development. Journal of molecular medicine. 2011;89(8):743–52. doi: 10.1007/s00109-011-0759-x 21509575 10.1007/s00109-011-0759-x PMC3152611

[34] Fukushima Y, Oshika Y, Tsuchida T, Tokunaga T, Hatanaka H, Kijima H, et al Brain-specific angiogenesis inhibitor 1 expression is inversely correlated with vascularity and distant metastasis of colorectal cancer. International journal of oncology. 1998;13(5):967–70. 9772287 10.3892/ijo.13.5.967

[35] Lee J, Koh J, Shin B, Ahn K, Roh J, Kim Y, et al Comparative study of angiostatic and anti-invasive gene expressions as prognostic factors in gastric cancer. International journal of oncology. 2001;18(2):355–62. 11172604

[36] Izutsu T, Konda R, Sugimura J, Iwasaki K, Fujioka T. Brain-specific angiogenesis inhibitor 1 is a putative factor for inhibition of neovascular formation in renal cell carcinoma. The Journal of urology. 2011;185(6):2353–8. doi: 10.1016/j.juro.2011.02.019 21511296 10.1016/j.juro.2011.02.019

[37] Nishimori H, Shiratsuchi T, Urano T, Kimura Y, Kiyono K, Tatsumi K, et al A novel brain-specific p53-target gene, BAI1, containing thrombospondin type 1 repeats inhibits experimental angiogenesis. Oncogene. 1997;15(18):2145–50. 9393972 10.1038/sj.onc.1201542

[38] Kudo S, Konda R, Obara W, Kudo D, Tani K, Nakamura Y, et al Inhibition of tumor growth through suppression of angiogenesis by brain-specific angiogenesis inhibitor 1 gene transfer in murine renal cell carcinoma. Oncology reports. 2007;18(4):785–92. 17786337

[39] Oka H, Chatani Y, Hoshino R, Ogawa O, Kakehi Y, Terachi T, et al Constitutive activation of mitogen-activated protein (MAP) kinases in human renal cell carcinoma. Cancer research. 1995;55(18):4182–7. 7664295

[40] Friday BB, Adjei AA. Advances in targeting the Ras/Raf/MEK/Erk mitogen-activated protein kinase cascade with MEK inhibitors for cancer therapy. Clinical Cancer Research. 2008;14(2):342–6. doi: 10.1158/1078-0432.CCR-07-4790 18223206 10.1158/1078-0432.CCR-07-4790

[41] Demuth HB, Beale MH, De Jess O, Hagan MT. Neural network design: Martin Hagan; 2014.

[42] LeCun Y, Bengio Y. Convolutional networks for images, speech, and time series. The handbook of brain theory and neural networks. 1995;3361(10):1995.

[43] Broad. Broad Institute TCGA Genome Data Analysis Center (2014): Analysis Overview for 15 July 2014. Broad Institute of MIT and Harvard. 2014.

[44] Love M, Anders S, Huber W. Differential analysis of RNA-Seq data at the gene level using the DESeq2 package. 2013.

[45] Friedman J, Hastie T, Tibshirani R. Regularization paths for generalized linear models via coordinate descent. Journal of statistical software. 2010;33(1):1 20808728 PMC2929880

[46] Masters T. Practical neural network recipes in C++: Morgan Kaufmann; 1993.

[47] Harrell FE, Lee KL, Mark DB. Tutorial in biostatistics multivariable prognostic models: issues in developing models, evaluating assumptions and adequacy, and measuring and reducing errors. Statistics in medicine. 1996;15:361–87. 8668867 10.1002/(SICI)1097-0258(19960229)15:4<361::AID-SIM168>3.0.CO;2-4

[48] Kanehisa M, Goto S. KEGG: kyoto encyclopedia of genes and genomes. Nucleic acids research. 2000;28(1):27–30. 10592173 10.1093/nar/28.1.27 PMC102409

