# Supplementary figures and images for: Cox-nnet: An artificial neural network method for prognosis prediction of high-throughput omics data
Source: PLoS Comput Biol. 2018 Apr 10;14(4):e1006076. doi: 10.1371/journal.pcbi.1006076 (PMC5909924; doi:10.1371/journal.pcbi.1006076)

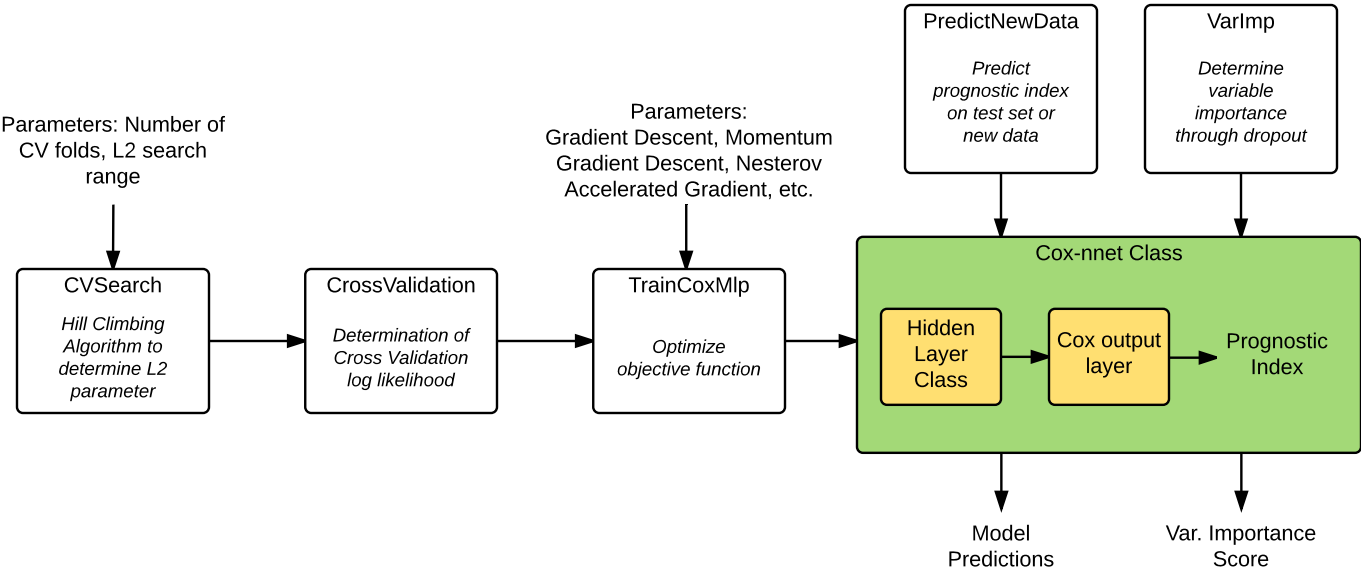

Supplement: S1 Fig — The arrows in the workflow point from each module to the output of that module. “Model Predictions” and “Var. Importance Score” are the output of Cox-nnet package. (PDF) [file pcbi.1006076.s001.pdf]

Performance across TCGA datasets (C-IPCW)

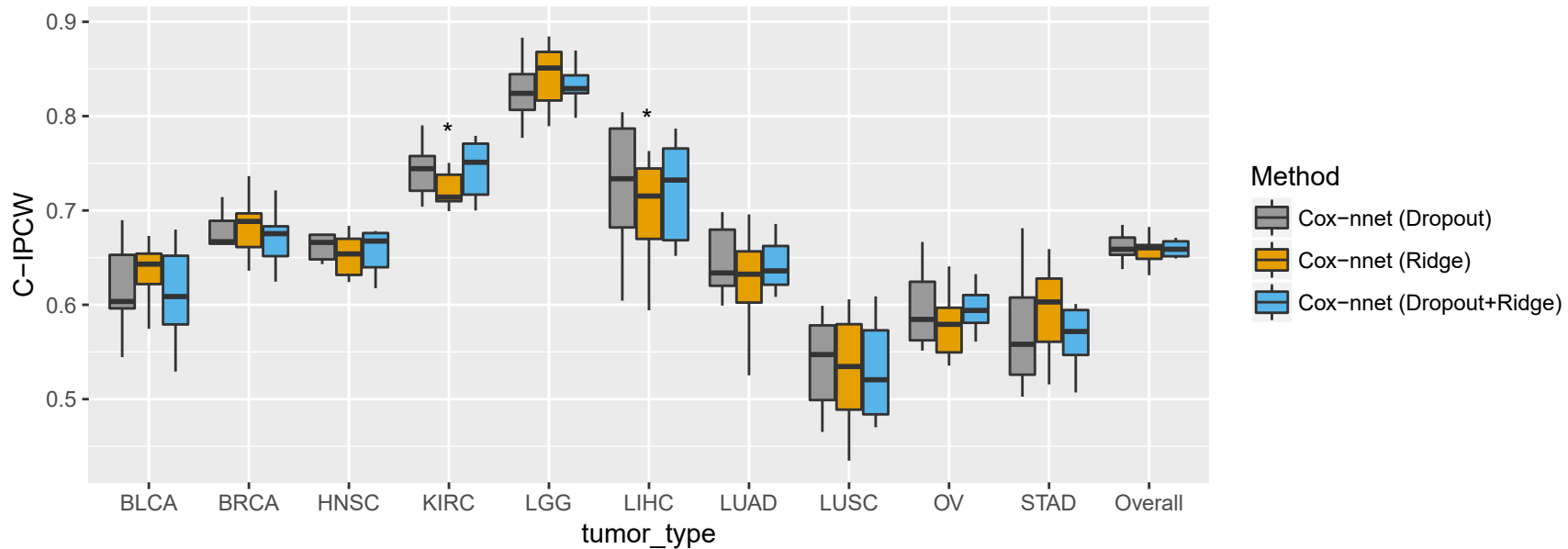

C-IPCW performance

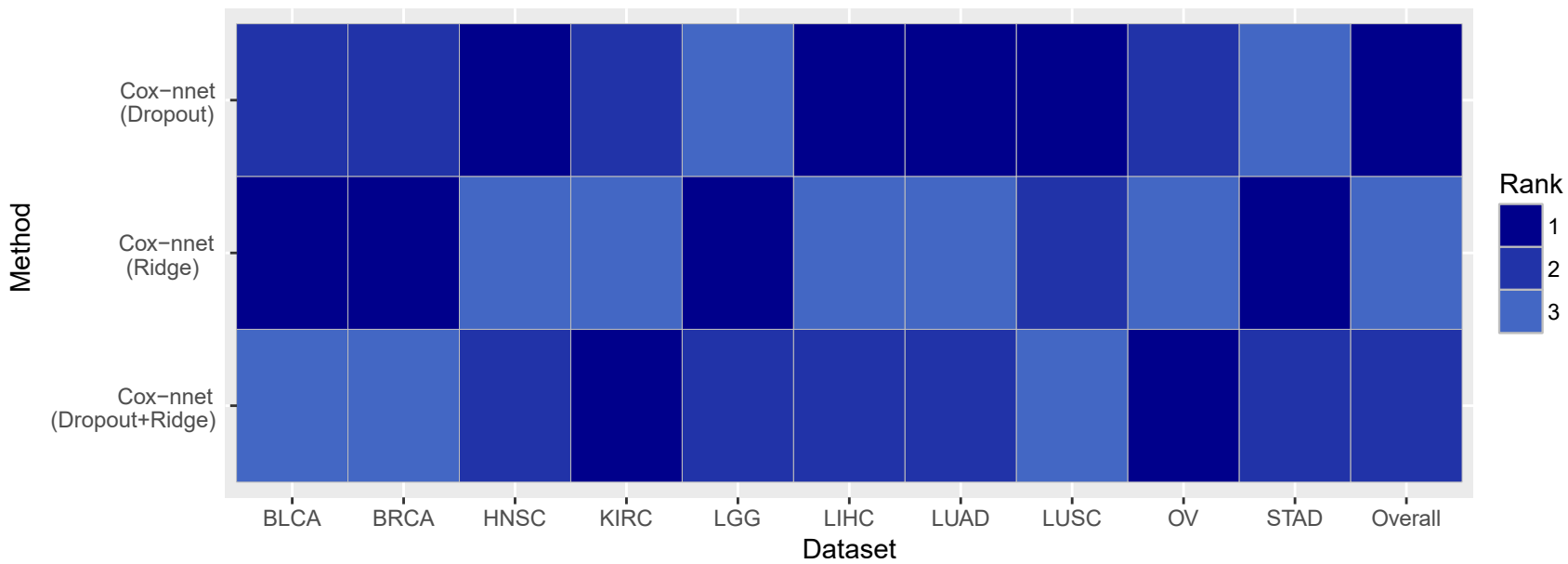

Supplement: S2 Fig — A. Boxplot of C-IPCW of the 10 TCGA datasets among various penalization approaches in Cox-nnet one hidden layer (ridge, drop-out and ridge combined with dropout). Cox-nnet with Ridge and Dropout is optimized based on 5-fold cross-validation. Cox-nnet parameterizations with Ridge and Dropout are optimized based on a single validation set. Each dataset is randomly split into 80% training and 20% testing sets and resampled 10 times to calculate the average performance of each approach. B. Performance rank of each regularization approach, ordered by their average performance in each dataset. (PDF) [file pcbi.1006076.s002.pdf]

Performance across TCGA datasets (C-IPCW)

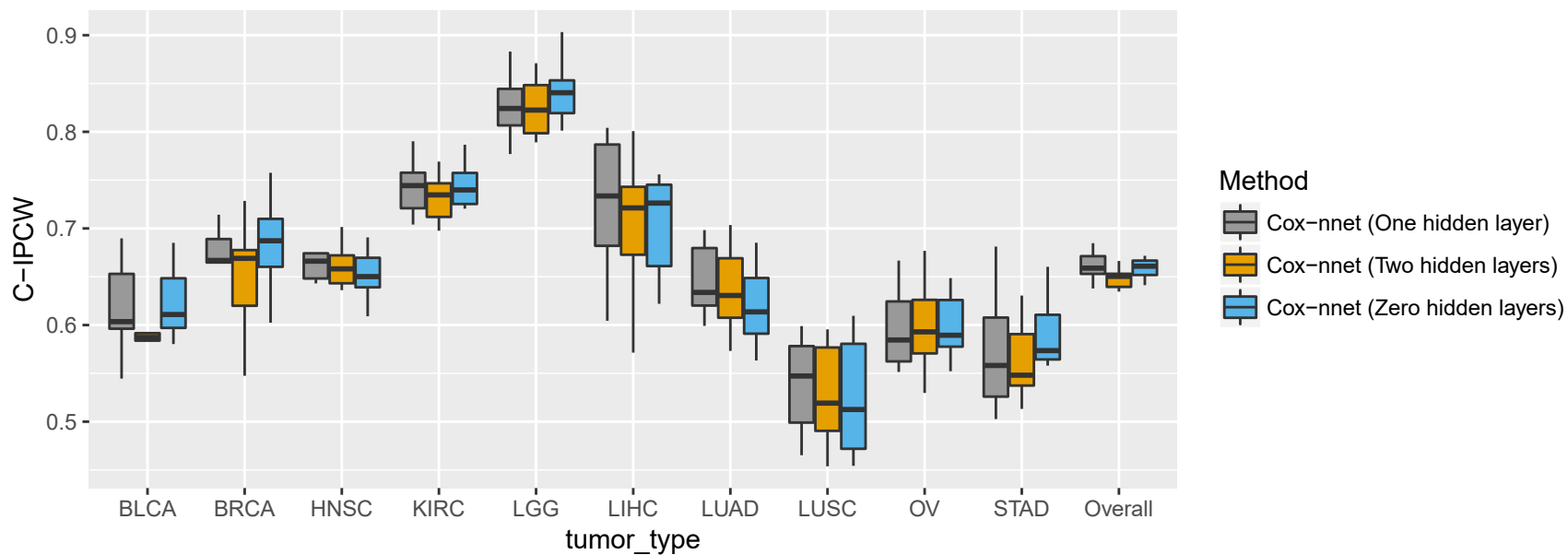

C-IPCW performance

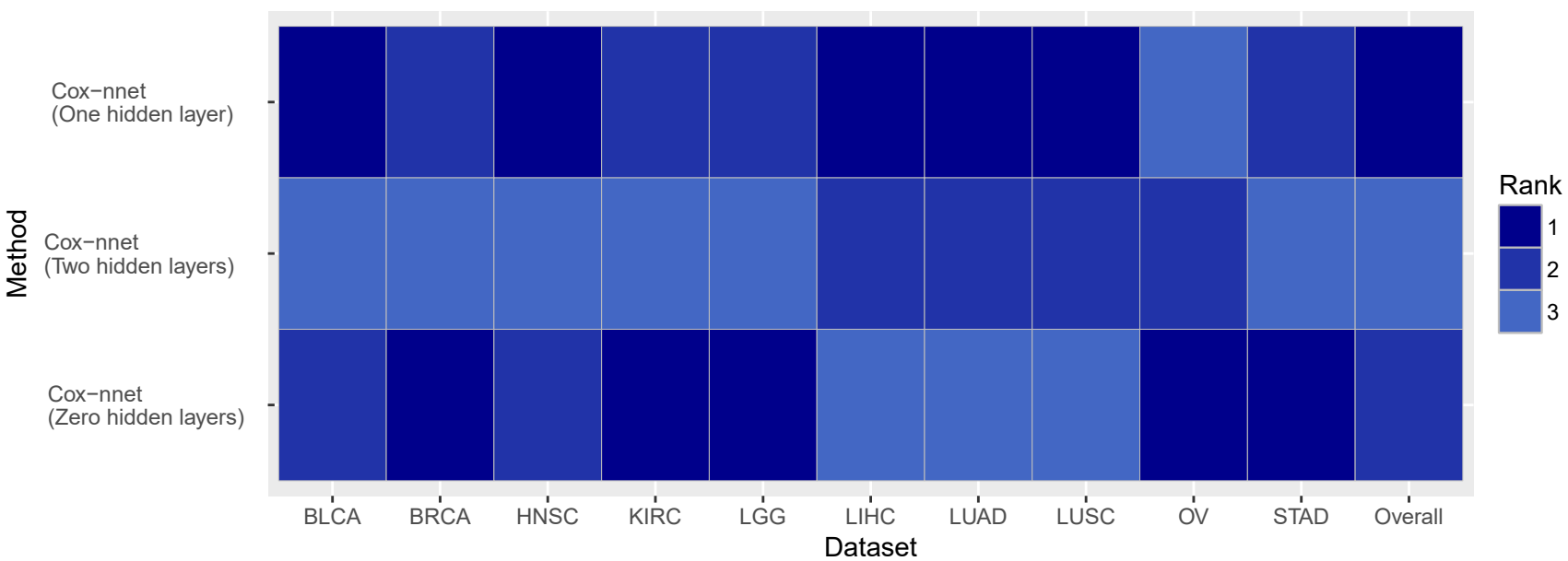

Supplement: S3 Fig — A. Boxplot of C-IPCW of the 10 TCGA datasets comparing zero, one and two hidden layers. Each model was optimized with 5-fold cross-validation. Each dataset is randomly split into 80% training and 20% testing sets and resampled 10 times to calculate the average performance of each approach. B. Performance rank of each regularization approach, ordered by the average C-IPCW scores in each dataset. *: P < 0.05. (PDF) [file pcbi.1006076.s003.pdf]

A

## Comparison of descent methods

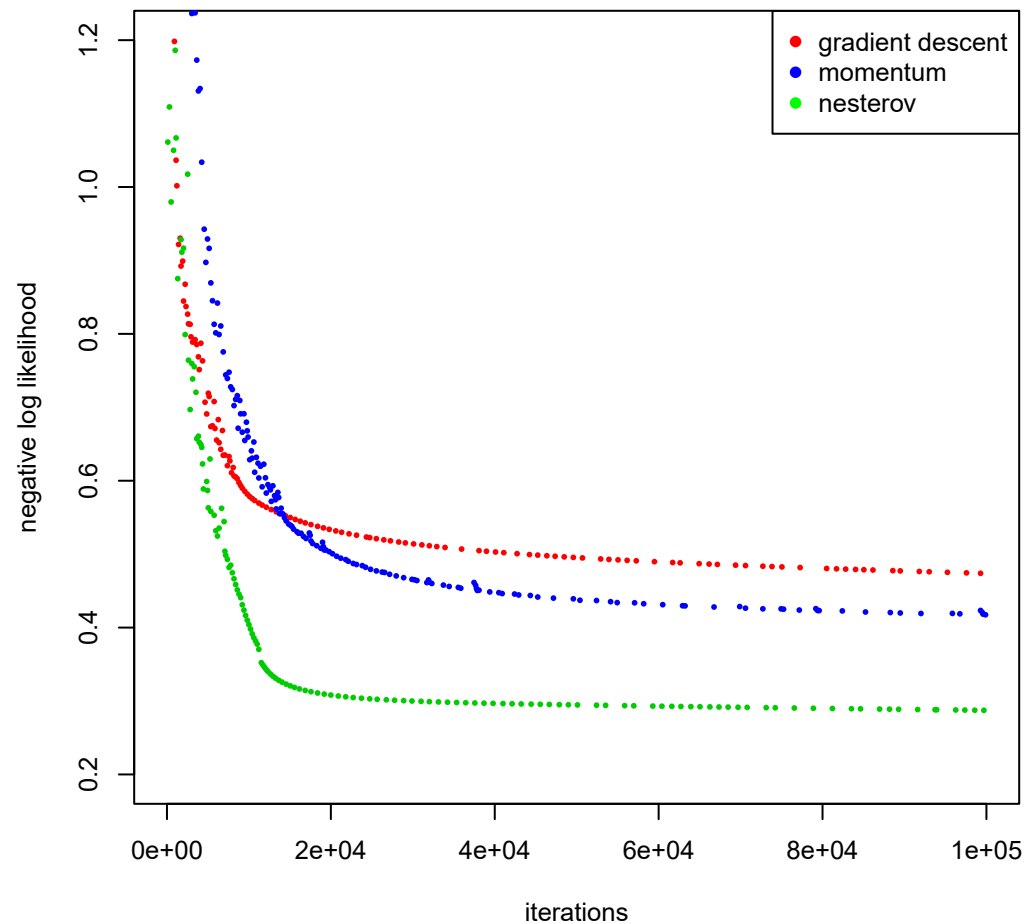

B

## Running time on KIRC dataset

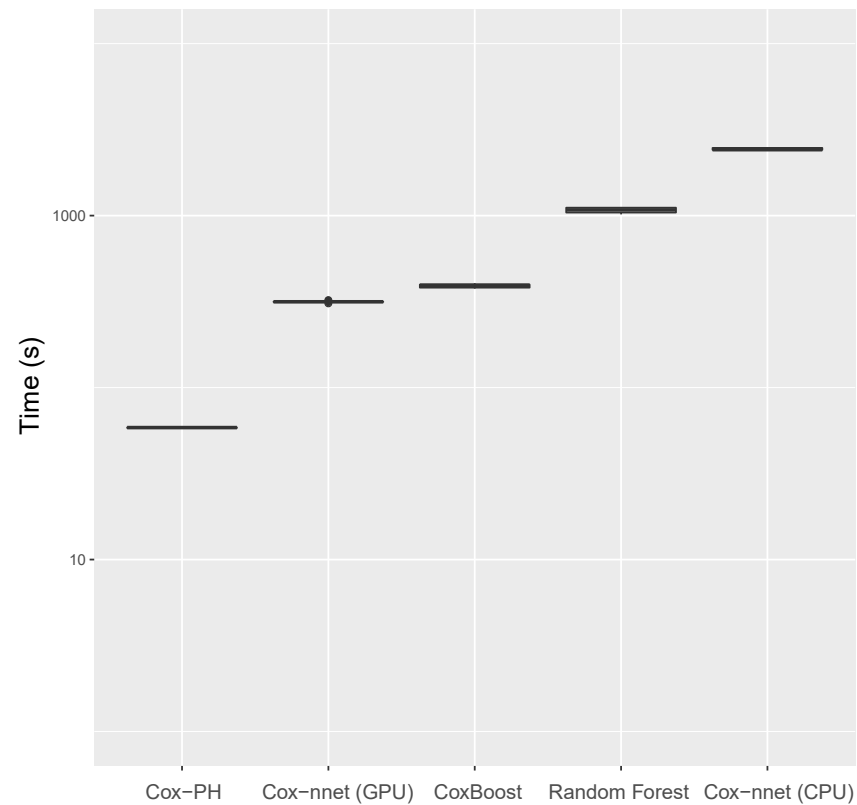

Supplement: S4 Fig — A: comparison of descent methods on the TCGA KIRC dataset. The change in cost function is evaluated over 100,000 iterations for three methods: gradient descent, momentum gradient descent and the Nesterov accelerated gradient. B: Boxplots comparing Cox-nnet (CPU and GPU), Cox-PH, Cox-boost and Random Forest (RS-F) running time on the same dataset. (PDF) [file pcbi.1006076.s004.pdf]

Performance across TCGA datasets (C-IPCW)

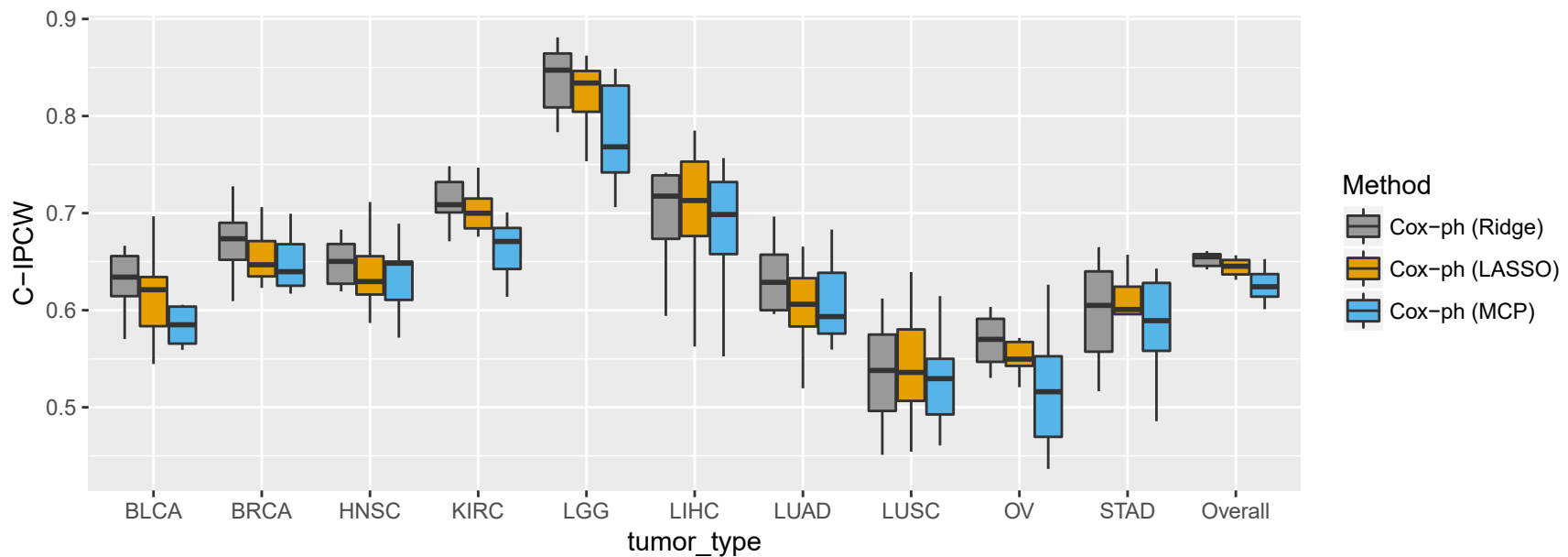

C-IPCW performance

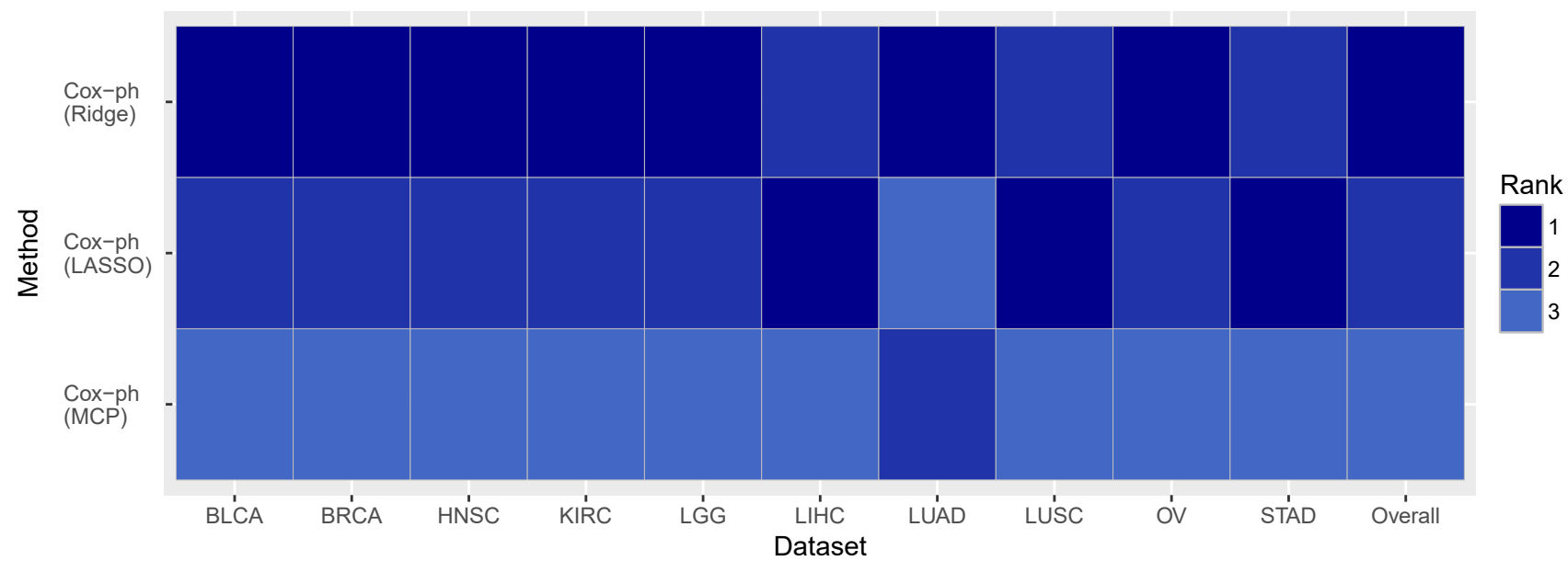

Supplement: S5 Fig — A. Boxplot of C-IPCW of the 10 TCGA datasets comparing Cox-PH regularization methods (LASSO, Ridge and MCP). Each model is optimized with 5-fold cross-validation. Each dataset is randomly split into 80% training and 20% testing sets and resampled 10 times to calculate the average performance of each approach. B. Performance rank of each regularization approach, ordered by the average C-IPCW scores in each dataset. *: P < 0.05. (PDF) [file pcbi.1006076.s005.pdf]

Performance across TCGA datasets (C-harrel)

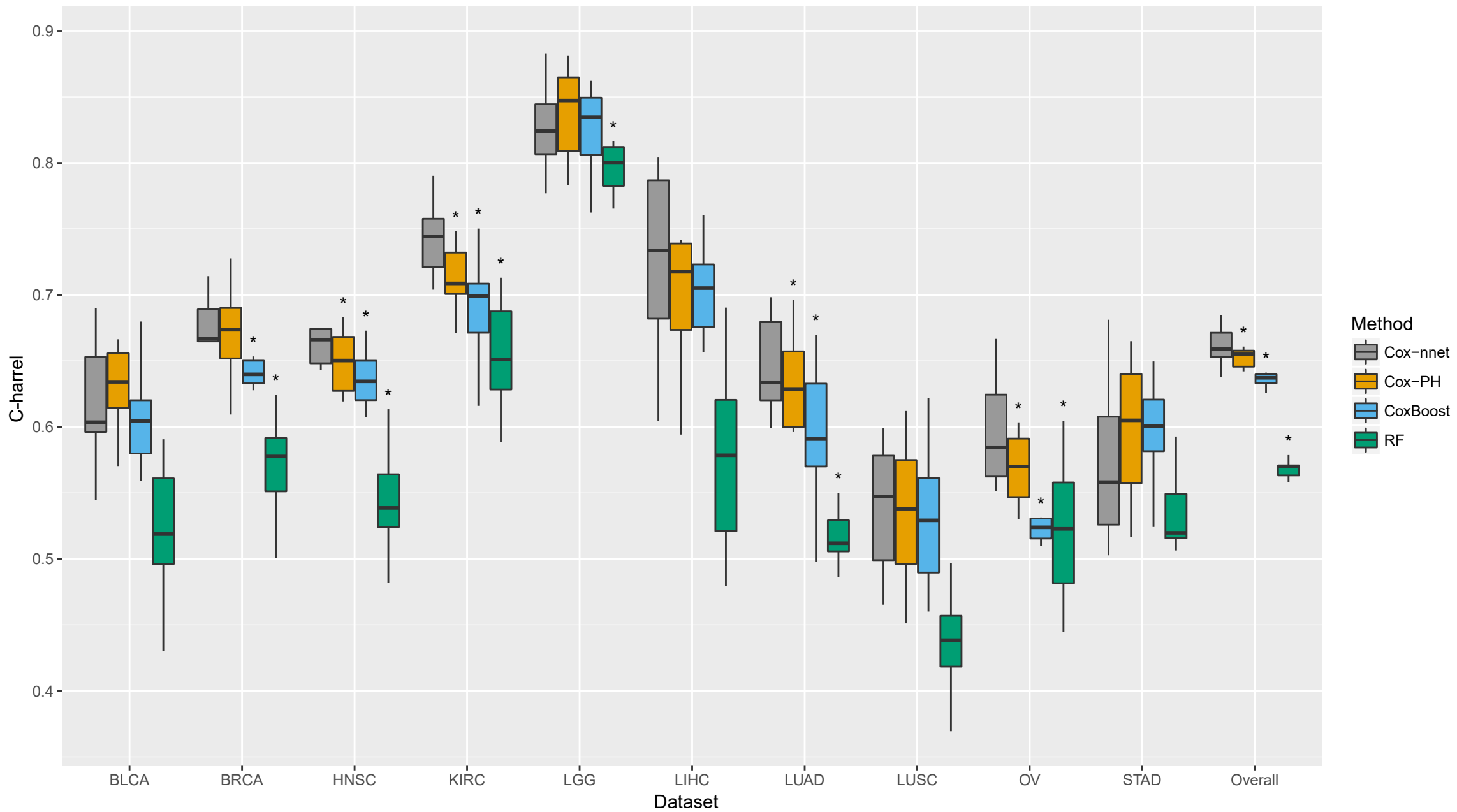

Supplement: S6 Fig — The data are randomly split into 80% training and 20% testing sets, and repeated 10 times to calculate the average C-harrel values in each approach. *: P < 0.05. (PDF) [file pcbi.1006076.s006.pdf]

A

Performance across TCGA datasets (Logrank)

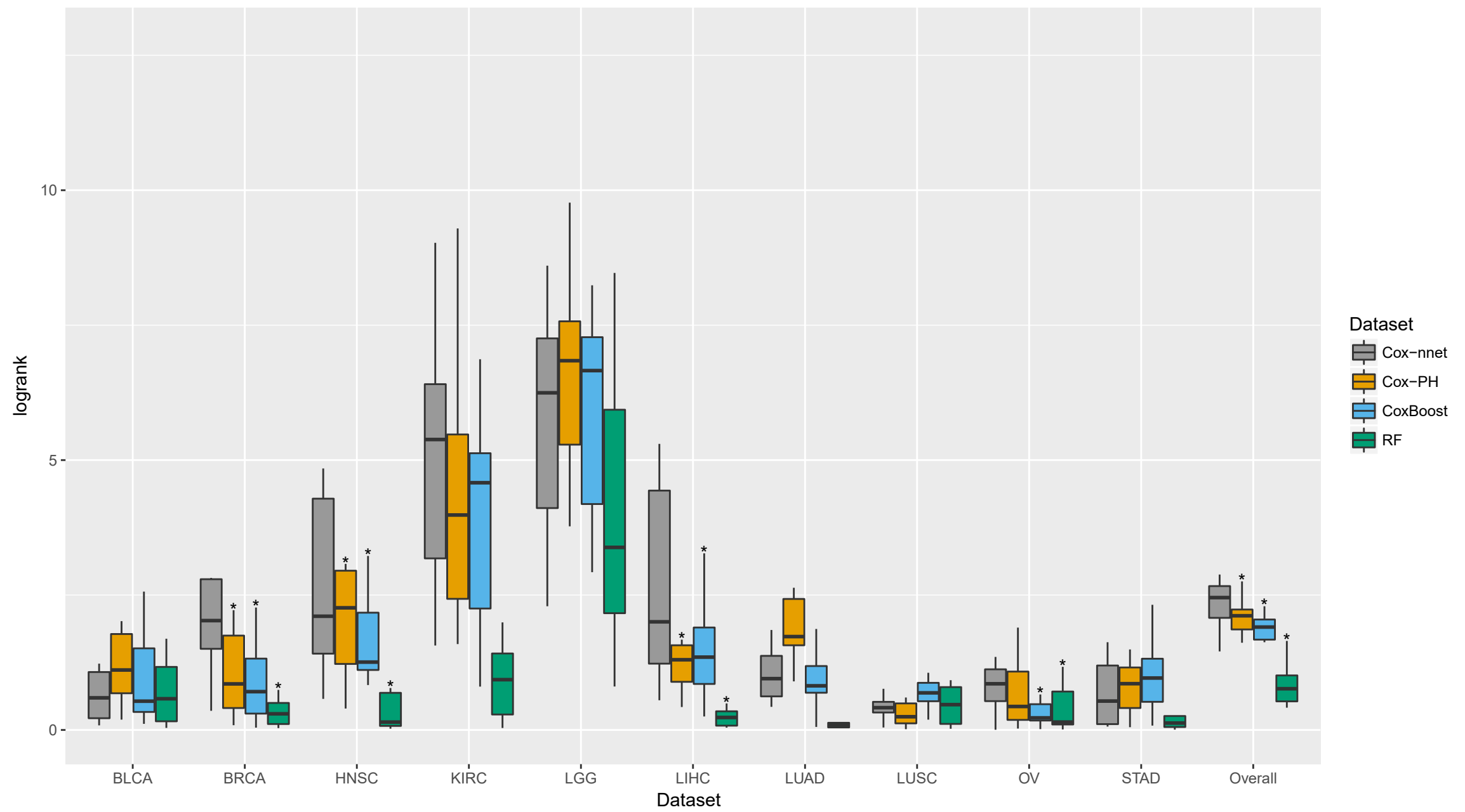

B

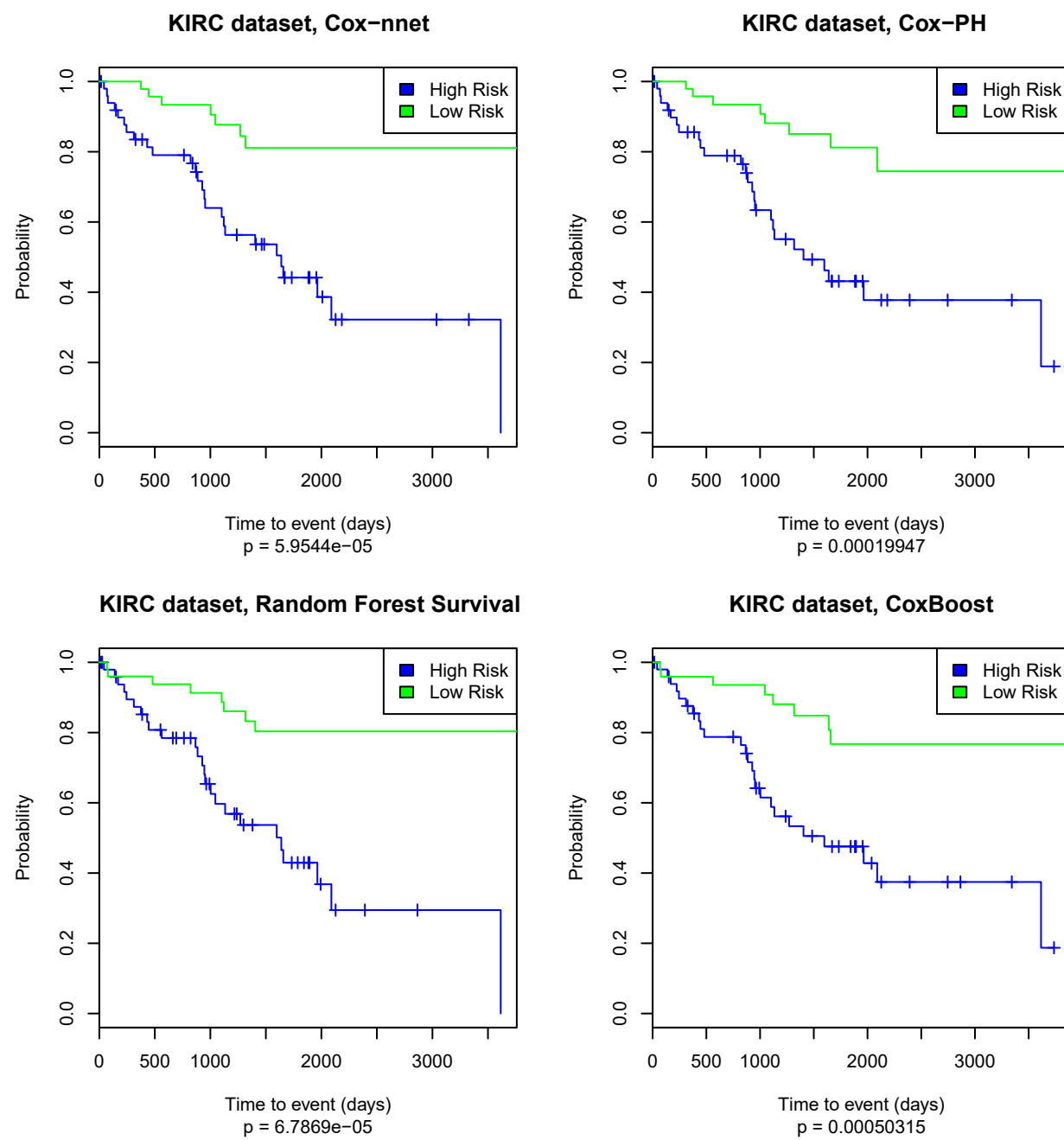

Supplement: S7 Fig — A. Bar plots of Log-rank p-values of the 10 TCGA datasets. The log rank p-values are calculated first splitting the patients by median prognostic index in the testing data set, in order to compare the survival distributions between the high and low risk groups. The data are randomly split into 80% training and 20% testing sets, and repeated 10 times to calculate the average log-rank p-values in each approach. *: P < 0.05. B. Kaplan-Meier plots from one exemplary repeat showing survival differences between the high and low risk groups. Note that due to dichotomization, log-rank p-values vary much widely compared to other performance metrics. (PDF) [file pcbi.1006076.s007.pdf]

Performance across TCGA datasets (Brier-score)

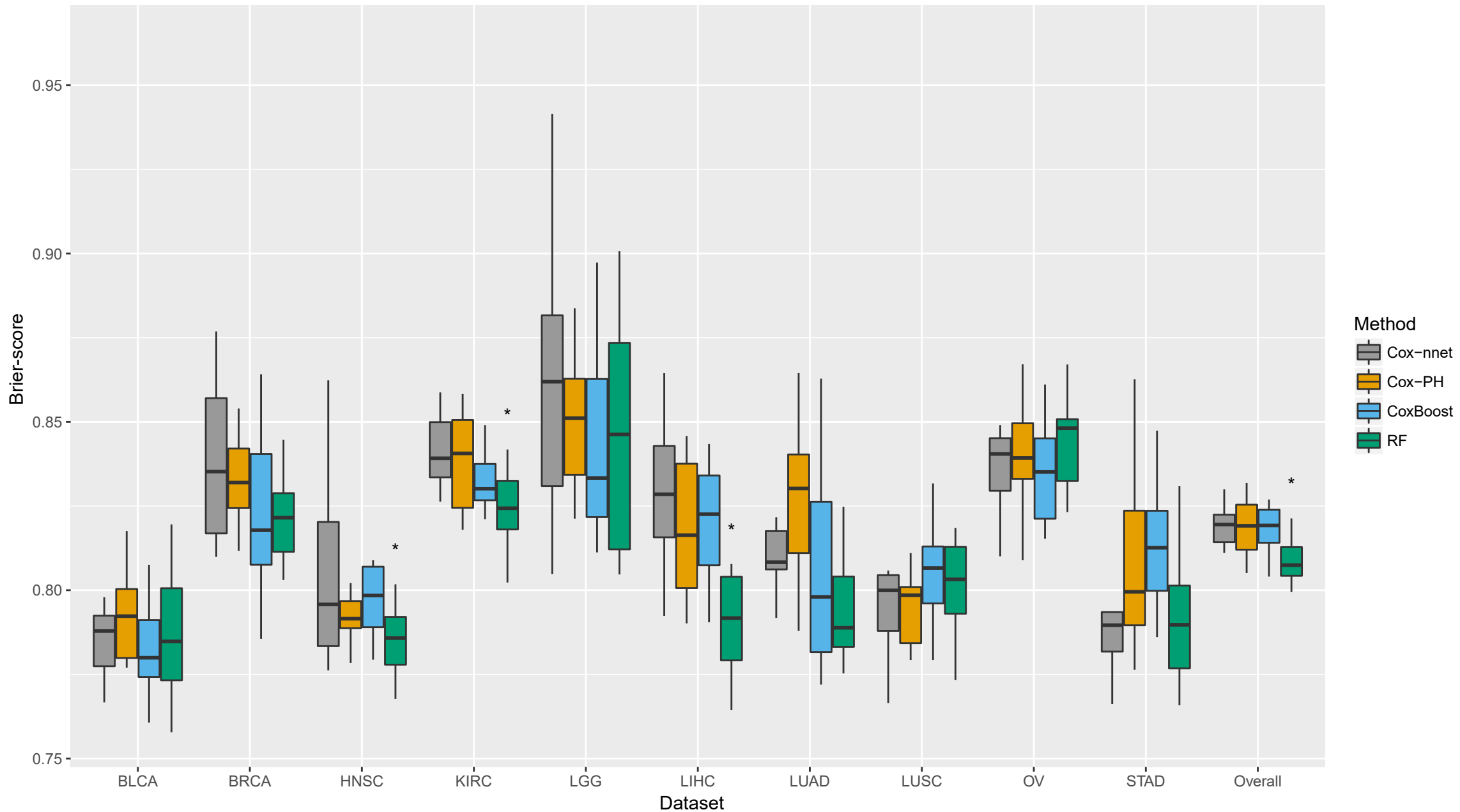

Supplement: S8 Fig — The data are randomly split into 80% training and 20% testing sets and repeated 10 times to calculate the average Brier scores in each approach. *: P < 0.05. (PDF) [file pcbi.1006076.s008.pdf]

# Selected genes from Cox-nnet enriched pathways

Cox-nnet Cox-PH

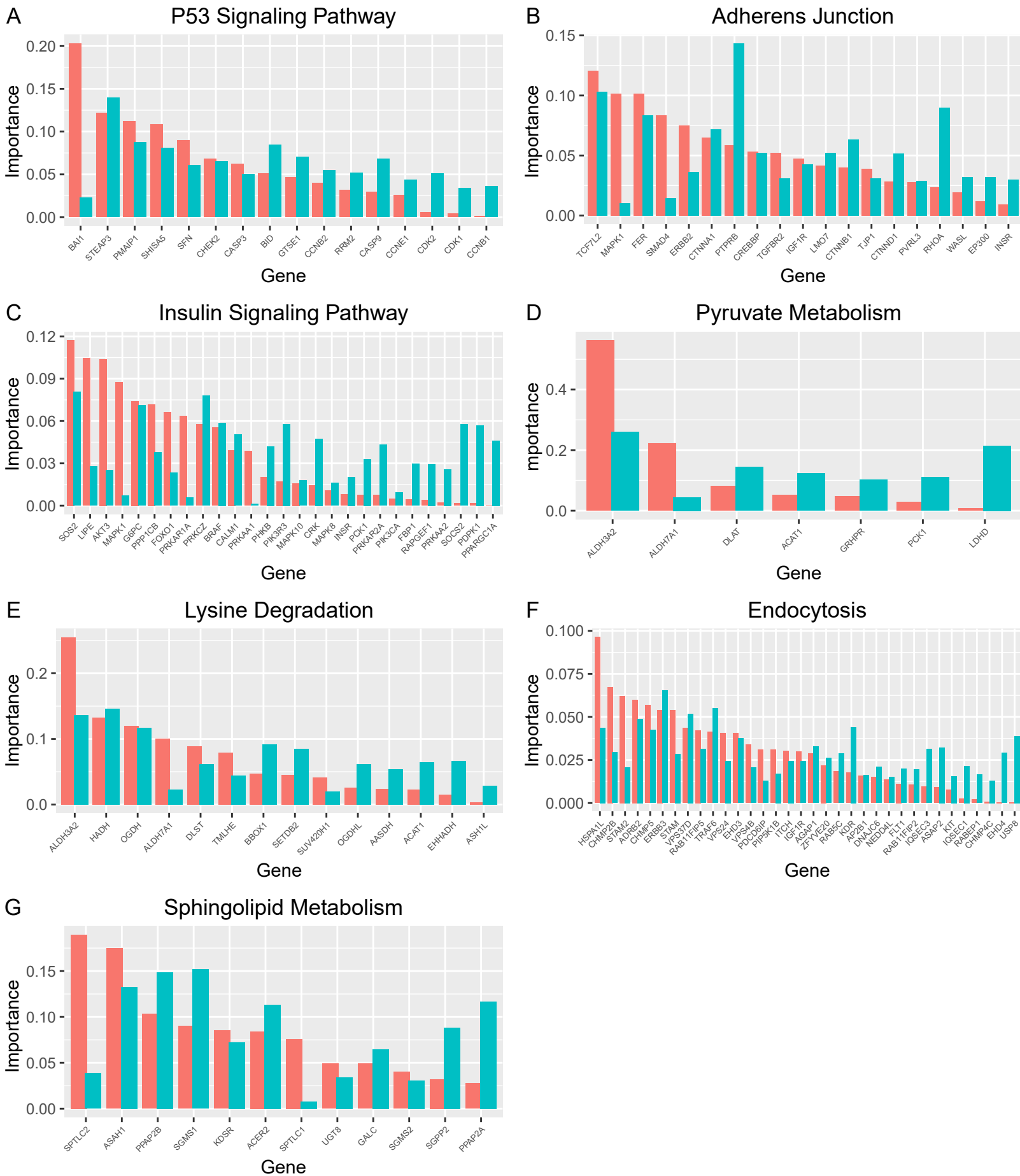

Supplement: S9 Fig — (PDF) [file pcbi.1006076.s009.pdf]

Simulation data metrics

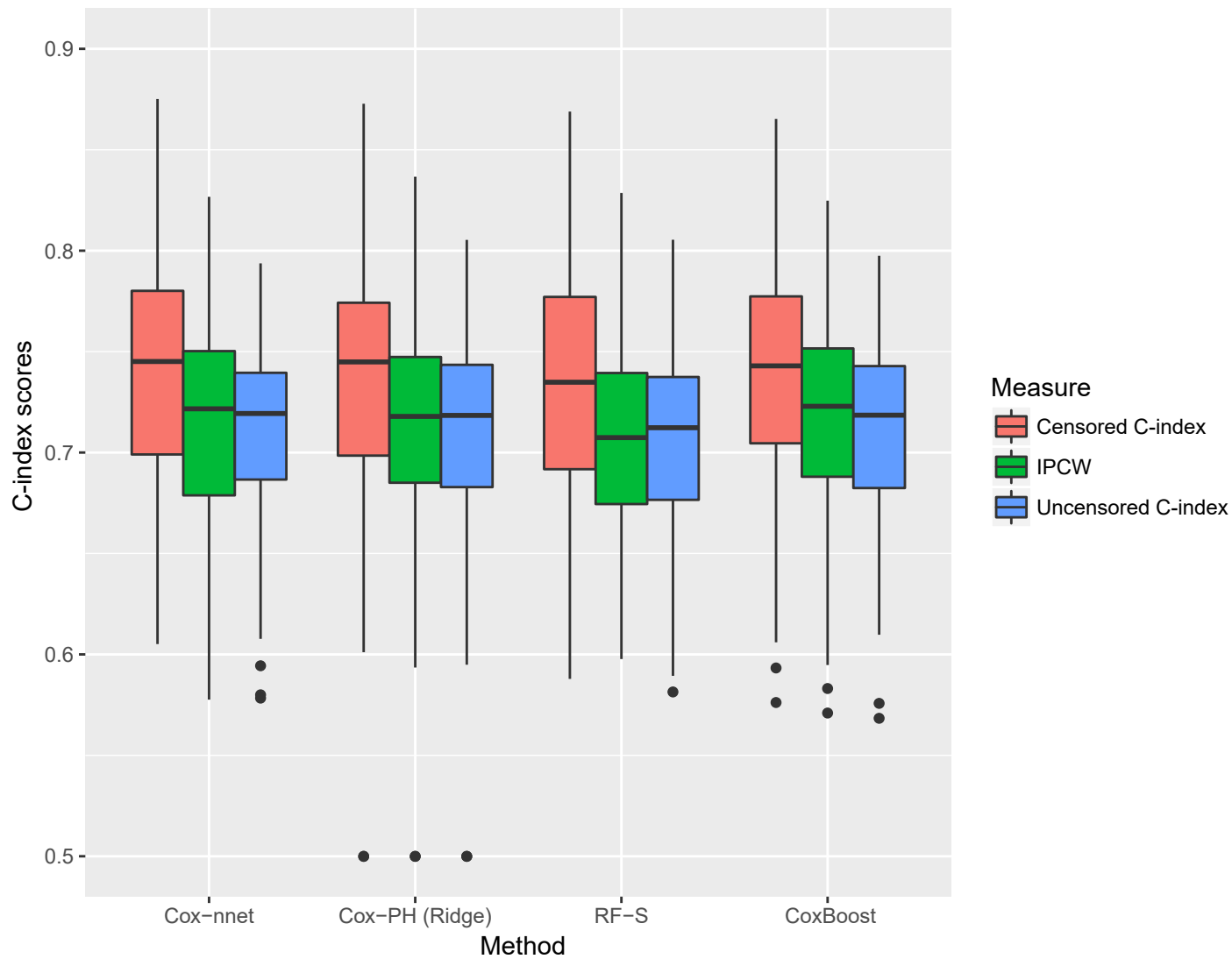

Supplement: S10 Fig — (PDF) [file pcbi.1006076.s010.pdf]
