# Supplementary material for: Cox-nnet: An artificial neural network method for prognosis prediction of high-throughput omics data
Source: PLoS Comput Biol. 2018 Apr 10;14(4):e1006076. doi: 10.1371/journal.pcbi.1006076 (PMC5909924; doi:10.1371/journal.pcbi.1006076)
Supplement: S2 Table — (PDF) [file pcbi.1006076.s012.pdf]

Cox-nnet node-associated enriched pathways

Significantly enriched pathways common for all nodes ($p < 0.05$)

Pathway	P.value	P.adjusted	Nodes
KEGG adherens junction	0.000	0.001	1-20
KEGG butanoate metabolism	0.000	0.001	1-20
KEGG cell cycle	0.000	0.001	1-20
KEGG citrate cycle tca cycle	0.000	0.001	1-20
KEGG cytokine cytokine receptor interaction	0.000	0.001	1-20
KEGG endocytosis	0.000	0.001	1-20
KEGG endometrial cancer	0.000	0.001	1-20
KEGG fatty acid metabolism	0.000	0.001	1-20
KEGG insulin signaling pathway	0.000	0.001	1-20
KEGG lysine degradation	0.000	0.003	1-20
KEGG p53 signaling pathway	0.000	0.003	1-20
KEGG peroxisome	0.000	0.001	1-20
KEGG ppar signaling pathway	0.000	0.001	1-20
KEGG propanoate metabolism	0.000	0.001	1-20
KEGG prostate cancer	0.000	0.001	1-20
KEGG proteasome	0.000	0.001	1-20
KEGG proximal tubule bicarbonate reclamation	0.000	0.001	1-20
KEGG pyruvate metabolism	0.000	0.001	1-20
KEGG renin angiotensin system	0.000	0.002	1-20
KEGG sphingolipid metabolism	0.001	0.005	1-20
KEGG systemic lupus erythematosus	0.000	0.001	1-20
KEGG tight junction	0.000	0.001	1-20
KEGG tryptophan metabolism	0.000	0.001	1-20
KEGG valine leucine and isoleucine degradation	0.000	0.001	1-20
KEGG vascular smooth muscle contraction	0.000	0.001	1-20

Additional significantly enriched pathways ($p < 0.05$)

Pathway	P.value	P.adjusted	Nodes
KEGG adipocytokine signaling pathway	0.001	0.005	1-19
KEGG beta alanine metabolism	0.000	0.003	1,3-20
KEGG colorectal cancer	0.000	0.001	1-13,15-20
KEGG homologous recombination	0.000	0.001	1-14,16-20
KEGG inositol phosphate metabolism	0.000	0.004	1-2,4-20
KEGG neurotrophin signaling pathway	0.000	0.001	1-13,15-20
KEGG nitrogen metabolism	0.001	0.007	1-16,18-20
KEGG phosphatidylinositol signaling system	0.000	0.003	1-2,4-20
KEGG aldosterone regulated sodium reabsorption	0.001	0.007	1-8,10-13,15-20
KEGG focal adhesion	0.000	0.001	1-13,16-20
KEGG mtor signaling pathway	0.002	0.012	1-5,7-13,15-20
KEGG renal cell carcinoma	0.000	0.001	1-13,15-18,20
KEGG ribosome	0.000	0.001	1-4,6-9,11-20
KEGG pathways in cancer	0.000	0.001	1-13,16-18,20
KEGG wnt signaling pathway	0.000	0.003	1-3,5-13,16-20
KEGG cytosolic dna sensing pathway	0.000	0.003	1-4,6-7,9,11-17,19-20
KEGG intestinal immune network for iga production	0.000	0.001	1-3,5,7-11,13-14,16-20
KEGG primary immunodeficiency	0.000	0.001	1-3,5,7-13,16-20
KEGG terpenoid backbone biosynthesis	0.002	0.011	1,3-5,7-13,16-20
KEGG tgf beta signaling pathway	0.000	0.001	1-13,17-18,20
KEGG vasopressin regulated water reabsorption	0.000	0.001	1-3,5,7-13,15,17-20
KEGG base excision repair	0.001	0.006	1-2,4-5,7-10,12-14,16-18
KEGG glycolysis gluconeogenesis	0.000	0.002	1,3-5,8,10-16,18-19
KEGG nod like receptor signaling pathway	0.000	0.001	2,5-7,9,11-16,19-20
KEGG erbb signaling pathway	0.000	0.003	1-4,8-12,17-18,20
KEGG pancreatic cancer	0.001	0.006	1-4,6,8,10-13,17-18
KEGG regulation of actin cytoskeleton	0.000	0.002	1-3,5-8,10,12-13,17-18
KEGG complement and coagulation cascades	0.000	0.001	4-5,7-9,11,14-16,19
KEGG dna replication	0.000	0.001	2,5,7,9,12-14,16,18-19

Pathway	P.value	P.adjusted	Nodes
KEGG glycosaminoglycan biosynthesis chondroitin sulfate	0.001	0.005	2,4,6,9,14-17,19-20
KEGG non small cell lung cancer	0.002	0.012	1,3-4,6,8,10-11,15,17
KEGG pathogenic escherichia coli infection	0.000	0.001	2,4,6-7,9,14-16,19
KEGG chronic myeloid leukemia	0.001	0.009	1,3,8,10-12,17-18
KEGG pyrimidine metabolism	0.000	0.004	2,4,6-9,14,17
KEGG thyroid cancer	0.006	0.023	1,3,5,8,10,12,17-18
KEGG graft versus host disease	0.000	0.001	7-9,11,16,19-20
KEGG leukocyte transendothelial migration	0.003	0.014	1,3,5,10,12-13,18
KEGG melanoma	0.005	0.023	1,3,8,10,12,17-18
KEGG arginine and proline metabolism	0.004	0.019	1,3-4,8,10,12
KEGG histidine metabolism	0.004	0.019	3-4,9,12,14,19
KEGG oxidative phosphorylation	0.000	0.001	1,5,10,12-13,19
KEGG long term potentiation	0.004	0.020	1,10,12,17-18
KEGG taste transduction	0.005	0.021	1-3,5,12
KEGG acute myeloid leukemia	0.012	0.041	4,8,10,17
KEGG allograft rejection	0.000	0.001	8-9,16,19
KEGG arrhythmogenic right ventricular cardiomyopathy arvc	0.011	0.038	2-3,7,18
KEGG glioma	0.006	0.025	1,8,10,17
KEGG leishmania infection	0.000	0.001	9,14,16,19
KEGG lysosome	0.000	0.002	1,3,10,12
KEGG natural killer cell mediated cytotoxicity	0.000	0.001	7,9,16,19
KEGG o glycan biosynthesis	0.008	0.031	1-2,6,17
KEGG parkinsons disease	0.005	0.020	1,5,10,12
KEGG spliceosome	0.001	0.007	6,11,14,17
KEGG calcium signaling pathway	0.003	0.015	1,12,18
KEGG gap junction	0.007	0.028	1,17-18
KEGG hematopoietic cell lineage	0.000	0.001	9,16,19
KEGG small cell lung cancer	0.005	0.022	8,10,17
KEGG toll like receptor signaling pathway	0.000	0.002	9,16,19
KEGG type i diabetes mellitus	0.000	0.001	9,16,19

Pathway	P.value	P.adjusted	Nodes
KEGG ubiquitin mediated proteolysis	0.006	0.026	1,10,17
KEGG amyotrophic lateral sclerosis als	0.012	0.041	6,19
KEGG antigen processing and presentation	0.000	0.001	16,19
KEGG autoimmune thyroid disease	0.000	0.002	16,19
KEGG chemokine signaling pathway	0.000	0.001	16,19
KEGG fc epsilon ri signaling pathway	0.013	0.043	4,17
KEGG huntingtons disease	0.004	0.020	1,19
KEGG jak stat signaling pathway	0.002	0.011	16,19
KEGG long term depression	0.006	0.024	1,17
KEGG melanogenesis	0.012	0.040	10,18
KEGG prion diseases	0.006	0.025	14,19
KEGG alzheimers disease	0.003	0.013	1
KEGG ascorbate and aldarate metabolism	0.003	0.016	4
KEGG asthma	0.003	0.016	19
KEGG basal cell carcinoma	0.006	0.023	4
KEGG cell adhesion molecules cams	0.006	0.024	19
KEGG drug metabolism cytochrome p450	0.012	0.040	4
KEGG fc gamma r mediated phagocytosis	0.004	0.017	19
KEGG glycine serine and threonine metabolism	0.009	0.033	4
KEGG glycosaminoglycan biosynthesis keratan sulfate	0.010	0.035	14
KEGG mismatch repair	0.016	0.050	7
KEGG primary bile acid biosynthesis	0.007	0.026	5
KEGG purine metabolism	0.012	0.041	14
KEGG steroid biosynthesis	0.008	0.029	19
KEGG t cell receptor signaling pathway	0.004	0.017	19
KEGG viral myocarditis	0.000	0.003	19
