# Supplementary material for: Cox-nnet: An artificial neural network method for prognosis prediction of high-throughput omics data
Source: PLoS Comput Biol. 2018 Apr 10;14(4):e1006076. doi: 10.1371/journal.pcbi.1006076 (PMC5909924; doi:10.1371/journal.pcbi.1006076)
Supplement: S3 Table — (PDF) [file pcbi.1006076.s013.pdf]

# GSEA enrichment of Cox-PH associated cancer pathways

## List of significantly enriched pathways ($p < 0.05$)

| Pathway | E.score | P.value | P.adjusted |
|---|---|---|---|
| KEGG cell cycle | -0.407 | 0.000 | 0.003 |
| KEGG citrate cycle tca cycle | 0.607 | 0.000 | 0.003 |
| KEGG fatty acid metabolism | 0.561 | 0.000 | 0.003 |
| KEGG lysosome | 0.410 | 0.000 | 0.003 |
| KEGG oxidative phosphorylation | 0.418 | 0.000 | 0.003 |
| KEGG proximal tubule bicarbonate reclamation | 0.633 | 0.000 | 0.003 |
| KEGG valine leucine and isoleucine degradation | 0.577 | 0.000 | 0.003 |
| KEGG olfactory transduction | 0.294 | 0.000 | 0.005 |
| KEGG cytokine cytokine receptor interaction | -0.330 | 0.000 | 0.005 |
| KEGG proteasome | -0.540 | 0.000 | 0.005 |
| KEGG vascular smooth muscle contraction | 0.391 | 0.000 | 0.005 |
| KEGG peroxisome | 0.415 | 0.000 | 0.006 |
| KEGG tight junction | 0.343 | 0.000 | 0.006 |
| KEGG propanoate metabolism | 0.518 | 0.001 | 0.009 |
| KEGG ppar signaling pathway | 0.406 | 0.001 | 0.011 |
| KEGG renin angiotensin system | 0.645 | 0.001 | 0.011 |
| KEGG systemic lupus erythematosus | -0.377 | 0.001 | 0.014 |
| KEGG prostate cancer | 0.368 | 0.002 | 0.016 |
| KEGG pathogenic escherichia coli infection | -0.433 | 0.003 | 0.034 |
| KEGG endometrial cancer | 0.409 | 0.004 | 0.040 |
| KEGG butanoate metabolism | 0.458 | 0.005 | 0.042 |
| KEGG epithelial cell signaling in helicobacter pylori infection | 0.361 | 0.006 | 0.047 |
| KEGG nod like receptor signaling pathway | -0.407 | 0.006 | 0.047 |
| KEGG beta alanine metabolism | 0.521 | 0.006 | 0.047 |
| KEGG complement and coagulation cascades | -0.393 | 0.007 | 0.047 |

| Pathway | E.score | P.value | P.adjusted |
|---|---|---|---|
| KEGG tryptophan metabolism | 0.427 | 0.007 | 0.047 |
| KEGG arginine and proline metabolism | 0.381 | 0.007 | 0.047 |
| KEGG calcium signaling pathway | 0.272 | 0.007 | 0.047 |
| KEGG spliceosome | -0.333 | 0.007 | 0.047 |
| KEGG parkinsons disease | 0.304 | 0.008 | 0.048 |
